# Adding Rare Earth Oxide Markers to Polyoxymethylene to Improve Plastic Recycling through Tracer-Based Sorting

**DOI:** 10.3390/polym16182591

**Published:** 2024-09-13

**Authors:** Aleksander Jandric, Christoph Olscher, Christian Zafiu, Robert Lielacher, Christoph Lechner, Andrea Lassenberger, Florian Part

**Affiliations:** 1 Department of Water-Atmosphere-Environment, Institute of Waste Management and Circularity, BOKU University, Muthgasse 107, 1190 Vienna, Austria; aleksander.jandric@boku.ac.at (A.J.); christian.zafiu@boku.ac.at (C.Z.); florian.part@boku.ac.at (F.P.); 2 Polymerwerkstatt GmbH, Dr.-Franz-Wilhelm-Straße 2, 3500 Krems an der Donau, Austria; rl@polymerwerkstatt.at (R.L.); cl@polymerwerkstatt.at (C.L.); 3 CEA Leti, 17 Avenue des Martyrs, 38054 Grenoble, France; andrea.lassenberger@cea.fr; 4 Xenocs SAS, 1–3 Allée du Nanomètre, 38000 Grenoble, France

**Keywords:** tracer-based sorting, plastic markers, rare earth oxides, polyoxymethylene, post-consumer plastic waste, plastic recycling, circular economy

## Abstract

Engineering plastics, such as polyoxymethylene (POM), are high-performance thermoplastics designed to withstand high temperature or mechanical stress and are used in electronic equipment, the automotive industry, construction, or specific household utensils. POM is immiscible with other plastics but due to a low volume of production, no methods were developed to separate it from the residual plastic waste stream. Therefore, POM recycling is minimal despite its high market value. This paper provides a proof of concept for tracer-based sorting (TBS) as a potential solution for increasing the separation efficiency of low-volume, high-quality polymers. For this purpose, yttrium oxide (Y_2_O_3_) and cerium (IV) oxide (CeO_2_) have been embedded into the POM matrix. Mechanical tests of samples at varying concentrations (0.1 to 1000 ppm) of both tracers were conducted, followed by an analysis of detectability and dispersibility using a portable X-ray fluorescence spectrometer (p-XRF), subsequently optimizing detection time and tracer concentration. Finally, an experimental scenario was developed to test the fate and potential recovery of the tracer material after the thermal treatment of plastics. A low detectable concentration, short measurement time, low influence on mechanical parameters of the compound, and low loss ratio after simulated recycling prove Y_2_O_3_ to be a suitable tracer for the industrial implementation of TBS.

## 1. Introduction

In 2021, annual global plastics production has surpassed 390 million tons. A total of 90.2% of this volume is estimated to be fossil-based, 8.3% is estimated to be post-consumer recycled plastics, and 1.5% is estimated to be bio-based/bio-attributed plastics 1. More than 10% of those produced plastics are so-called engineering plastics, which are a subcategory of thermoplastics. They are designed and modified using functional fillers to withstand different conditions compared to commodity plastics [1]. Those conditions can include high temperature, high load or shear stress, highly acidic or basic conditions, and others [2]. Engineering plastics are usually produced in a lesser volume than commodity plastics, leading to a lower percentage of engineering plastic waste within waste streams. As there is a wide variety of different types of plastic, sorting and recycling is very challenging because it requires very efficient sorting methods (e.g., automated sensor-based technologies) to separate the different plastics into uniform waste streams.

Blending distinct plastic types inevitably results in a decline in quality, ultimately culminating in downcycling or thermal utilization. This is because technical parameters, such as the shear strength of the recycled material, often undergo a negative transformation compared to the virgin material. If those deviations are too severe, recycled plastics may be excluded from certain usages [3]. Publications and reports have already highlighted the need for more efficient sorting systems to separate plastic waste into homogeneous (single origin) waste streams to achieve the recycling goals, which are in line with, for example, the European Strategy for Plastics in a Circular Economy and the Circular Economy Action Plan of the European Union [1,4,5,6,7]. The most advanced techniques for the management of plastic waste currently in use are sorting systems that employ a variety of sophisticated technologies. These include optical sensors for color separation and near-infrared (NIR) spectroscopy for identifying thermoplastics, such as high-density polyethylene (HDPE), low-density polyethylene (LDPE), polypropylene (PP), polyethylene terephthalate (PET), polystyrene (PS), or polyvinyl chloride (PVC). In addition, swim–sink processes based on density separation are used in plastics recycling plants. Nevertheless, these state-of-the-art sorting techniques currently available are unable to detect, separate, and concentrate engineered plastics, such as polyoxymethylene (POM), which has found application in the automotive sector, construction sector, and different household items, mainly based on its high mechanical resistance. For this reason, most engineered plastics are not currently recycled, resulting in the majority of these materials being incinerated instead. To find a technical solution to this global problem, various research groups have developed the so-called tracer-based sorting (TBS) concept [8,9,10,11,12]. The modification of thermoplastics by incorporating special marker substances (e.g., upconverting nanoparticles) enables the separation and production of homogeneous, high-quality plastic types, thereby enhancing recycling efficiency through TBS plants [9,13,14]. The first applications of TBS were tested on different plastic packaging materials (e.g., PET bottles and their labels), but it is now being applied to a broader range of polymers using different molecules like organic dyes, lanthanide markers, and engineered nanomaterials [8,9,15]. In our previous work [16], a detailed review of the spectroscopic methods used for plastic sorting was given, including a list of marker candidates that can be used for TBS. Furthermore, the review encompasses the technology readiness level (TRL) and details the strengths and weaknesses of the different TBS methods, which are still in the developmental phase and not yet implemented on an industrial scale.

This study demonstrates the proof of concept of two marker candidates, identified and described in more detail in our previous work [16], which can be incorporated into the polymer matrix during extrusion. Since many recycling plants are already equipped with spectroscopy-based sensor systems, the focus lies on such detection methods. For the laboratory-scale experiments, yttrium oxide (Y_2_O_3_) and cerium (IV) oxide (CeO_2_) particles were added to POM during extrusion. Both markers are conventional, REACH-registered rare earth particles, thus authorized for the EU market. In line with the circular economy, CeO_2_ could alternatively be sourced from, e.g., glass polishing waste [17]. The objective was to test the influence of the markers on technical parameters, as well as their detectability in different concentrations using X-ray fluorescence (XRF), for which high-throughput sorter systems would already be available on the market. Two recycling scenarios were considered and assessed. For the “closed-loop recycling” scenario, the XRF detection time was optimized. Given the current relevance of waste incineration of engineered plastics (“thermal treatment” scenario), we assessed the potential recovery of the marker substances, which can be concentrated and found in the solid residues (ashes) after incineration.

## 2. Materials and Methods

**Figure 1:**
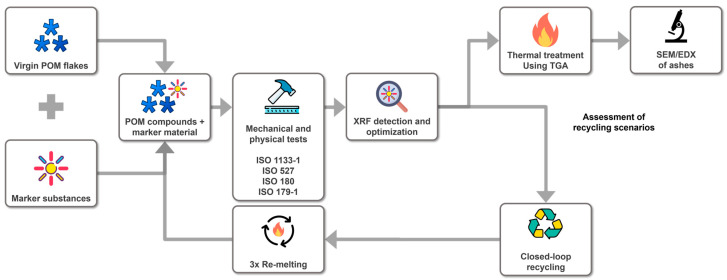
Experimental design and workflow to produce and detect ytterbium oxide (Y_2_O_3_) and cerium oxide CeO_2_ markers in polyoxymethylene (POM). “Closed-loop recycling” was assessed by re-melting the POM pellets with the marker materials, while “thermal treatment” was simulated using thermogravimetric analysis (TGA).

Plastic pellets were produced by adding CeO_2_ (CAS No. 1306-38-3, particle size: 13–20 µm, China, imported by Polymerwerkstatt GmbH, Krems, Austria) and Y_2_O_3_ (CAS No. 1314-36-9, particle size: 4–6 µm, China, imported by Polymerwerkstatt GmbH, Krems, Austria) powders to POM homo- and co-polymers during extrusion (Section 2.1). The pellets were melted to create test specimens, which were used to test the mechanical properties (Section 2.2), determine the crystallinity and phase transitions (Section 2.3), and evaluate the detectability of the marked plastics (Section 2.4) to demonstrate the proof of concept regarding closed-loop recycling (Section 2.5.1). Figure 1 summarizes the experimental design and workflow to demonstrate the proof of concept regarding the “closed-loop recycling” and “thermal treatment” scenarios.

### 2.1. Production of POM Composites with Marker Substances

All pellets were made with the POM homo-polymer and the POM co-polymer using the twin-screw extruder of the plastic compounder Polymerwerkstatt GmbH in Krems, Austria. It is noted that a homo-polymer refers to a polymer where only one monomer is used for polymerization, whereas in a co-polymer, multiple monomers are chained to a polymer, with none of the monomers exceeding more than 95% of the polymer composition [18]. The POM co-polymer used was HOSTAFORM^®^ C 13021, abbreviated POM C 13 R. The POM homo-polymer used was a DELRIN^®^ 500 acetal homo-polymer, abbreviated POM H-500. To create a masterbatch of either the homo-polymer or co-polymer, the virgin POM flakes were mixed with the respective marker substance in the concentration of 3333 mg/kg or 5000 mg/kg. The final concentration series was produced through serial dilution of the base mixtures, whereby the final concentrations of CeO_2_ or Y_2_O_3_ are 0.1, 1, 10, 100, and 1000 ppm. The production of the compound started with the mixing of the marker substance or pellets of the previous concentration with virgin POM flakes. The resulting mixture was introduced into a twin-screw extruder with eight heating elements, which gradually heated the mixture at a pressure of ~20 bar from 106 °C to 215 °C and pushed it through a forming screen at the end of the extruder nozzle. The resulting POM filaments were cooled using a cooling bath consisting of water at room temperature (RT) and processed into pellets using an automatic chopper. The test specimens were manufactured from pellets using an injection molding process according to specified dimensions as per ISO-179-1 [19].

### 2.2. Mechanical Test

To test the mechanical properties of POM plastics containing the marker substance, standardized tests of mechanical properties were performed using sample pellets and test specimens of the highest (1000 ppm) and lowest (0.1 ppm) concentration of each marker in the composite as well as control samples without marker substances for both the homo- and co-polymer. All material tests are based on guidelines of the International Organization for Standardization (ISO) to enable a comparison of the POM test objects with and without the marker material. This includes ISO 1133-1 [20] to determine the melt mass flow rate (MFR) and melt volume flow rate (MVR) of thermoplastics using sample pellets, ISO 179-1 [19] to evaluate the Charpy impact properties using test specimens, and ISO 527 [21,22] to determine the tensile properties using test specimens.

### 2.3. Small- and Wide-Angle X-ray Scattering Measurements

Small- and wide-angle X-ray scattering (SAXS, WAXS) was used to observe the influence of the marker material on the lamellar phase dimensions and crystallinity within the composite pellets. Measurements were performed on a Nano-InXider (Xenocs, Grenoble, France) using a Genix 3D micro-focus X-ray source with 2D single reflection multilayer optics, providing clean Cu $\kappa_{\alpha }$ radiation at 8.04 KeV and 1.54 Å wavelength. The beam path is windowless and fully evacuated from beam delivery to detector; the beam is collimated with a scatterless pinhole system providing high flux and low background beam. SAXS and WAXS data are continuously detected by a Pilatus 3 200 k (SAXS) and 100 k (WAXS) hybrid pixel detector (Dectris AG, Baden, Switzerland) at a fixed sample-to-detector distance of 0.937 m (SAXS) and 0.08 m (WAXS), covering a *q*-range from 0.002 < q and Å^−1^ < 0.4, with the scattering vector defined as $q=\frac{4\pi }{\lambda }sin\theta ,\;$with λ being the X-ray wavelength and *θ* defined as the scattering angle between the incident and scattered beam. Samples were self-standing and fixed on the solid’s holder. Two-dimensional data are automatically corrected for dark and cosmic radiation by the Nano-InXider data acquisition software. One-dimensional data are normalized from solid angle, exposure time, and transmitted intensity. Data are normalized by sample thickness during background subtraction to obtain data on an absolute scale. XSACT 2.10.2 software (Xenocs, Grenoble, France) was used to treat and analyze SAXS and WAXS data. Data were azimuthally averaged and subtracted from the empty beam to result in a typical scattering curve. Information on the lamellar phase of the polymer was extracted by correlation function analysis of the Fourier-transformed SAXS scattering data with the Strobl model [23,24]. With this method, the long and short periods of the polymer, as well as the degree of crystalline fraction, can be extracted. WAXS two-dimensional data were azimuthally averaged and plotted. It is noteworthy that no anisotropy was observed.

### 2.4. Portable X-ray Fluorescence (pXRF)

#### 2.4.1. Detection of Markers in Polymer Composites

**Figure 2:**
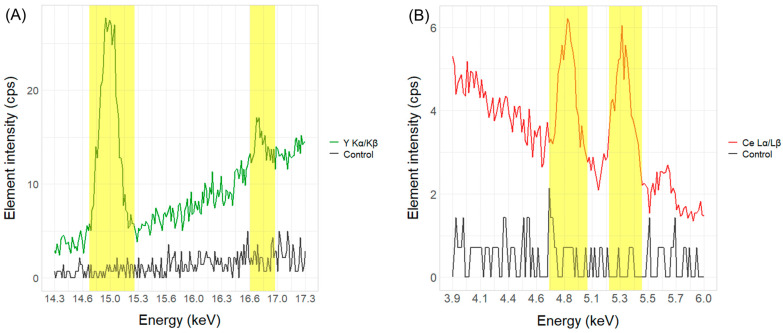
(**A**) The XRF spectrum section with yttrium Kα (highlighted area at 14.958 kEV) and Kβ (highlighted area at 16.738 kEV) peaks at 100 ppm concentration (green line) in the POM plastics compared to the control sample without marker substance (black line). (**B**) The XRF spectrum section with cerium Lα (highlighted area at 4.4840 keV) and Lβ (highlighted area at 5.262 keV) peaks at 1000 ppm concentration (red line) in the POM plastics compared to the control sample (black line).

The technology with the highest technology readiness level (TRL) for industrial-scale sorting of waste plastic materials relies on X-ray fluorescence detection or infrared detection (IR) [16]. The following detection and quantification of marker substances were performed with a portable X-ray fluorescence spectrometer (pXRF) XL3T950 (Thermo Scientific Portable Analytical Instruments Inc., Tewksbury, USA) using the manufacturer’s default software program “Environmental mode”. The equipment was calibrated using experimentally derived calibration calculations (Section 2.4.2). All measurements were conducted in a measuring chamber to ensure additional radiation isolation and reduce interfering factors, like the change in the angle between the radiation source and the sample, changes in the measuring position on the sample, and overall consistency. The XRF source was a gold anode, with the tube’s maximum voltage being 50 kV and maximum current being 100 μA. Based on the working principle of the pXRF, the target element present in the sample will have characteristic α and β peaks clearly distinguishable from the background. For detection of the Y_2_O_3_ marker, the optimal positions in the spectrum are yttrium characteristic Kα and Kβ peaks at 14.958 and 16.738 keV, respectively. For detecting the CeO_2_ markers, potential positions in the pXRF spectrum are Kα and Kβ peaks at 34.720 and 39.257 keV, respectively, as well as Lα and Lβ peaks at 4.840 and 5.262 keV, respectively. However, during the pilot testing of POM plastics with the CeO_2_ marker substance, Lα and Lβ were shown to be significantly more reliable across different measurement times. Therefore, all further calculations were based on the cerium peaks at those positions in the spectrum. An example of the yttrium Kα and Kβ and cerium Lα and Lβ peaks in POM plastics compared to the zero samples without the marker substance is shown in Figure 2.

#### 2.4.2. Calibration of pXRF for Y_2_O_3_ and CeO_2_ Marker Substances

Separate calibrations for homo- and co-polymer POM samples containing Y_2_O_3_ and CeO_2_ in concentrations of 0, 0.1, 1, 10, 100, and 1000 ppm were calculated. POM samples containing Y_2_O_3_ and CeO_2_ at benchmark concentrations of 100 and 1000 ppm, respectively, were measured in time intervals of 1 s, 5 s, 10 s, 20 s, 30 s, 40 s, and 50 s. The elemental peak saturation was observed at a 30 s measurement time (see Figure S1 in the Supplementary Materials). These results coincide well with the results of other studies on pXRF analysis of plastic additives [25,26].

**Figure 3:**
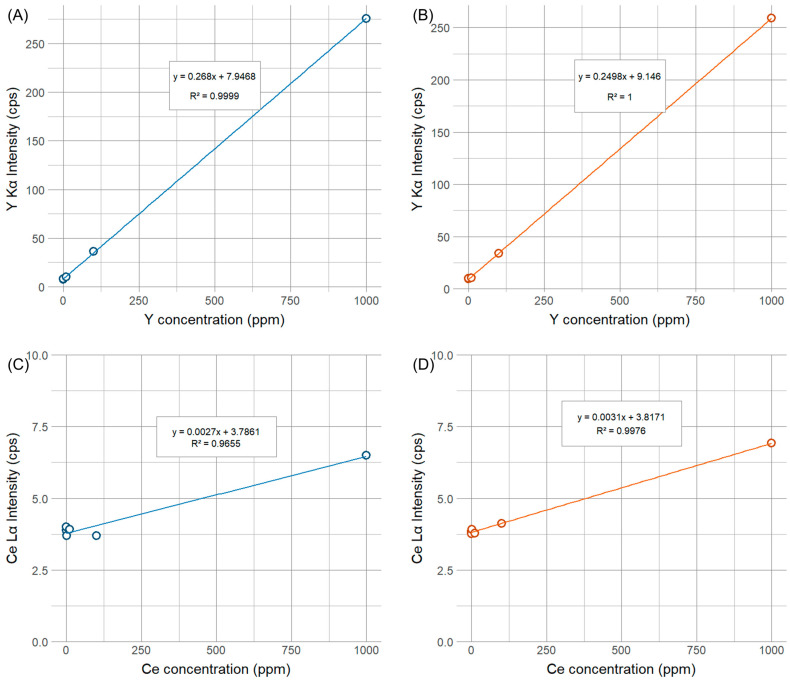
Calibration linear regression models for both marker substances in POM homo- and co-polymers at 0, 0.1, 1, 10, 100, and 1000 ppm concentrations. Graph (**A**) depicts the calibration line for the Y_2_O_3_ marker in the POM homo-polymer; graph (**B**) depicts the Y_2_O_3_ marker in the POM co-polymer, graph (**C**) depicts the CeO_2_ marker in the POM homo-polymer, and graph (**D**) depicts the CeO_2_ marker in the POM co-polymer.

The calibration curves for the marker substances and polymer types were developed by measuring each concentration aliquot five times without moving the sample for 30 s (see Figure 3). A summary of the Y Kα and Ce Lα measurements corresponding to the different marker concentrations is shown in Table S1 in the Supplementary Materials. The signal limit of detection (LOD) has been calculated using the following equation:

(1) $$LOD=S_{reag}+3\sigma_{reag}$$

S_reag_—signal mean value of the blank sample;

σ_reag_—standard deviation of the blank signal;

LOD—limit of detection.

The R^2^ adjusted goodness of fit for Y Kα is 0.9999 for both polymer types, while the R^2^ adjusted goodness of fit for Ce Lα for the homo-polymer is 0.9976 and 0.9655 for the co-polymer. Furthermore, from the pXRF measurements of the calibration aliquots at a 30 s measurement time and a 100 ppm Y_2_O_3_ concentration, the determined precision of the pXRF device was 0.67 cps (1.92 ppm) and 1.46 cps (4.25 ppm) for the homo- and co-polymer, respectively. The precision of the pXRF device at a 30 s measurement time and a 1000 ppm CeO_2_ concentration corresponds to 0.52 cps (74.46 ppm) and 0.38 cps (58.55 ppm) for the homo- and co-polymer, respectively.

#### 2.4.3. Evaluation of Dispersibility

For the dispersibility test, the experimental setup consisted of 10 co-polymer test specimens and 10 homo-polymer test specimens containing the Y_2_O_3_ marker, as well as 10 co-polymer test specimens and 10 homo-polymer test specimens containing the CeO_2_ marker. Each sample was analyzed with the pXRF for 30 s in three positions per sample plate, i.e., on both ends and in the middle of the test specimen. Each position was analyzed in three repetitions.

For the Y_2_O_3_ marker, a concentration of 100 ppm was selected as the benchmark concentration since the 10 ppm concentration was too close to the LOD for the pXRF device. For the CeO_2_ marker, a concentration of 1000 ppm was chosen, as pXRF measurements indicated lower sensitivity for this marker substance.

After pXRF measurements of the sample set, a single ANOVA was conducted separately for the homo- and co-polymers containing both markers individually. The aim was to test whether the variation in the Y Kα peak height for the Y_2_O_3_ marker and the Lα peak for the CeO_2_ marker within a single test specimen was higher than the variation between different test specimens of the same polymer type.

### 2.5. Recycling Scenario Analysis

Two experimental scenarios were designed to test the marker substance fate in waste POM plastics: the “closed-loop recycling scenario” (Section 2.5.1) and the “thermal treatment scenario” (Section 2.5.2). The thermal treatment scenario simulates the most common formal waste treatment route by subjecting POM samples to thermogravimetric analysis (TGA) and analyzing the combustion residues. The closed-loop recycling scenario is simulated by re-melting and re-extruding the POM pellets containing marker substance with analysis of samples before and after the simulated recycling process.

#### 2.5.1. Closed-Loop Recycling Scenario

To simulate a common plastic recycling process, marked polymer pellets were subjected to three additional melting and extrusion cycles, simulating the re-melting of recycled plastic. The repetition of the melting and extrusion cycles followed the same process for the production of new POM pellets. In order to test for changes in mechanical properties after the simulated recycling process, five tests following standards ISO 527-2 (tensile properties) [22], ISO 180 (Izod impact strength) [27], and ISO 1133 (MFR and MVR) [20] were performed on sample pellets and test specimens containing 100 ppm of the Y_2_O_3_ marker substance after initial production and after threefold repeated re-melting and extrusion cycles.

Triplicate 30 s pXRF measurements using the “Environmental mode” were conducted for homo- and co-polymer sample pellets before (control sample group) and after threefold repeated re-melting and extrusion cycles (experimental group) to test the impacts of the simulated recycling process on marker substance concentration. The two sample sets before and after the simulated recycling process included 10 POM pellets each for the homo- and co-polymer, containing 100 ppm of the Y_2_O_3_ marker substance.

#### 2.5.2. Thermal Treatment Scenario

POM pellets containing the Y_2_O_3_ marker at a concentration of 100 ppm, as well as a control group without marker substances, were subjected to a TGA. Approximately 1 g of the marked POM pellets were weighed using an analytical balance (SARTORIUS, CP225D-0CE, LOD = 0.01 mg) and placed in an aluminum oxide crucible with an inner diameter of 1.5 cm. The TGA was performed using a Netzsch STA 409 C/CD thermal analyzer (Erich Netzsch B.V. & Co. Holding KG, Selb, Germany) with argon and oxygen as flow-through gases. At the beginning, a warm-up phase gradually increased the temperature of the sample to 34 °C over a period of 10 min. The following program heated the sample to 500 °C with a heating rate of 2 °C per minute (detailed protocol overview available in Tables S2–S4 in the Supplementary Materials). This program was chosen after earlier tests with the material, resulting in spontaneous combustion of the sample and the clogging of the gas vents in the analyzer. The resulting ash was stored at room temperature in a small glass beaker and was sealed with a plastic lid and parafilm to ensure no material was lost during transport.

The TGA sample residues were analyzed using a scanning electron microscope (SEM) (Apreo 2 SEM [Thermo Fisher Scientific Inc., Waltham, MA, USA] and FEI Quanta 200 SEM [FEI Company, Hillsboro, OR, USA]) coupled with energy-dispersive X-ray spectroscopy (EDX) (Octane Elect Plus EDX platform) [AMETEK Inc., Berwyn, PA, USA]). The combustion residues were mounted using a carbon adhesive strip on an aluminum sample carrier. The SEM subjects the samples to a high vacuum (pressure of 10^−3^ to 10^−7^ mbar) and works with an energy of 20 keV. Images were taken at different magnifications (100×, 500×, 1000×, 10,000×). Regions of interest (ROIs) were defined and subjected to EDX analysis, which provided elemental composition and atom percentage of the ROIs. Unfortunately, the non-conductive nature of the polymer and the degradation of the samples through the high energy of the electron beam prevented elemental mapping of the surface of the test specimen.

## 3. Results

### 3.1. Mechanical Testing of POM Virgin Material Containing Marker Substance

Evaluating the MFR and MVR allows for the filler content, additive content, and bonding structure of polymers to be determined since both values correlate with the molecular weight of the polymer [28]. The summary and comparison of the MFR and MVR for POM homo- and co-polymer-containing marker substances are shown in Table S5 in the Supplementary Materials.

Further mechanical tests according to standards ISO 527-2 [22] and ISO 179-1 [19] were conducted on POM homo- and co-polymers containing both marker substances. Table 1 and Table 2 show the results of material tests on the POM co- and homo-polymer tests. Samples containing the marker substances show similar results across different mechanical tests compared to the control group (without marker substance), independent of the marker concentration. Looking at the strain at yield and the elongation at break, the control sample of the co-polymer (Table 1) shows a much higher percentage compared to the samples containing the markers. No significant differences were found between the homo-polymer samples with and without the markers. More details on the differences are discussed in Section 4.

### 3.2. SAXS and WAXS Results

**Figure 4:**
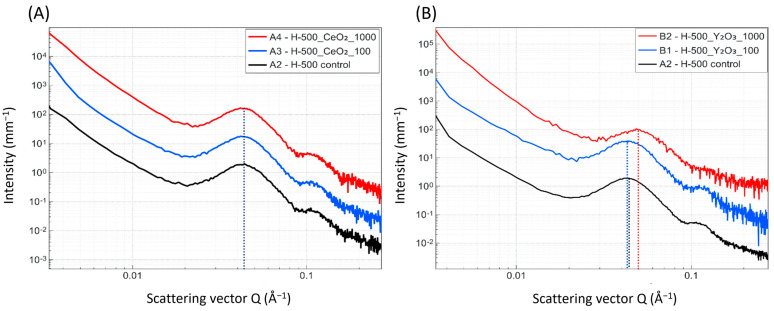
SAXS patterns of the POM homo-polymer (H500) with (**A**) CeO_2_ and (**B**) Y_2_O_3_ markers at different concentrations. The curves show a typical pattern of the lamellar structure of a semicrystalline polymer. Adding CeO_2_ does not influence the long period of the polymer structure. Y_2_O_3_ alters the lamellar structure.

Incorporating CeO_2_ nanoparticles into the polymer has only a minor influence on the lamellar phase dimensions. However, the total crystallinity extracted from correlation function analysis (Figure S2 in the Supplementary Materials) shows a gradual decrease from 0.20 to 0.11 with increasing CeO_2_ quantity (Table S6 in the Supplementary Materials). The influence of adding Y_2_O_3_ particles, in turn, is much more pronounced (Figure 4), resulting in a significative decrease in the short period (crystalline domains of the polymer) and, interestingly, an initial increase in the long period (100 ppm) with a following significant decrease in the long period. The crystallinity of these mixtures is increased upon the addition of Y_2_O_3_ (Table S6 in the Supplementary Materials), likely serving as nucleation sites during the cooling process. Polymer lamellar phases are very sensitive to the quenching process. However, the parameters of the cooling process are kept constant for samples with and without markers and can be excluded as the origin of variations in the lamellar structure.

**Figure 5:**
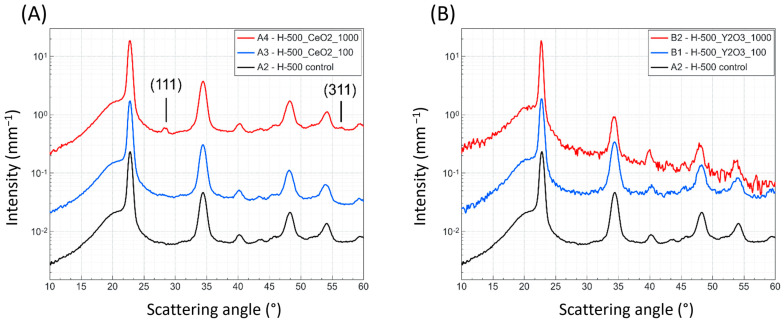
WAXS patterns of the POM homo-polymer (H500) with CeO_2_ and Y_2_O_3_ markers at different concentrations. Data show that the addition of the markers does not alter the position of the polymer peaks. (**A**) CeO_2_ can be detected at a concentration of 1000 ppm, visible in the 111 and 311 reflections of CeO_2_ at 28.6° and 56.6° 2θ, respectively. (**B**) Y_2_O_3_ does not show any additional peak.

WAXS measurements show a typical pattern of a semicrystalline polymer (Figure 5). None of the peak positions are influenced by the addition of CeO_2_ or Y_2_O_3_. CeO_2_ markers are detected at 1000 ppm and are visible in two additional peaks at 28.6° and 56.6° 2θ, corresponding to 111 and 311 reflections, respectively [29]. Y_2_O_3_ was not detected in the WAXS pattern at any concentration, likely due to the lower scattering power of Y_2_O_3_ compared to CeO_2_.

### 3.3. Marker Detectability Using XRF

**Figure 6:**
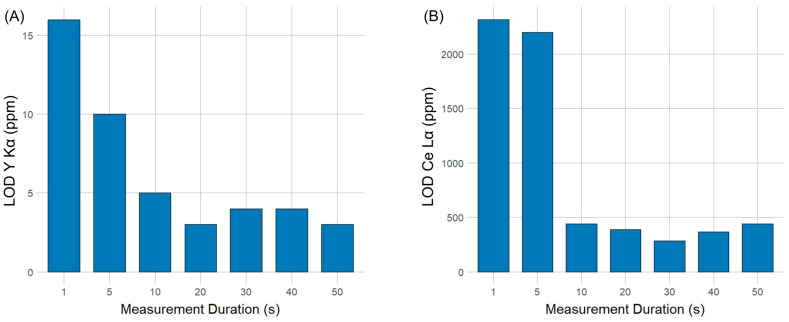
Calculated limit of detection (LOD) from calibration curves depending on the measurement time ranging between 1 and 50 s for the (**A**) Y_2_O_3_ and the (**B**) CeO_2_ markers.

To test the relationship between measurement time and marker concentration in POM plastics, a set of five samples (five separate POM pellets) with concentration levels ranging from 0 ppm to 1000 ppm were measured for 1, 5, 10, 20, 30, 40, and 50 s. For each measurement time, a single ANOVA in combination with the one-sided Dunnett’s post hoc test was performed to find the minimal measurement time for marker detection, enabling the determination of the limit of detection (LOD). The results of the calculated LODs are shown in Figure 6.

In the case of yttrium markers, the relationship between pXRF measurement time and Y_2_O_3_ concentrations was assessed by comparing the characteristic Y Kα peaks resulting from pXRF analysis. The LOD was calculated using the formula described in Section 2.4.2. and ranged from 3 cps (16 ppm) for a 1 s measurement to 8 cps (3 ppm) for a 50 s measurement (Figure 6). A one-sided Dunnett’s post hoc test was used to determine the concentration at which the Y Kα peak is significantly different from the control group, with a α ≤ 0.05 at the significance level. As shown in Table S7 in the Supplementary Materials, the concentration of the Y_2_O_3_ marker could be significantly distinguished from the control decreases with an increasing pXRF measurement time, ranging from a measurement time of 1 s for 100 pm to 10 s for 10 ppm, which is also the pXRF device’s absolute LOD, as increasing measurement times did not improve lower detection limits.

In the case of cerium markers, the LOD was calculated to range from 3 cps (2318 ppm) for a 1 s measurement to 4 cps (442 ppm). An overview of the relationship between the measurement time and LOD is shown in Figure 6. A one-sided Dunnett’s post hoc test showed that only the CeO_2_ marker concentration was significantly different from the control group at a 1000 ppm concentration with a minimal measurement time of 5 s (see Table S8 in the Supplementary Materials).

### 3.4. Dispersibility of the Markers

**Figure 7:**
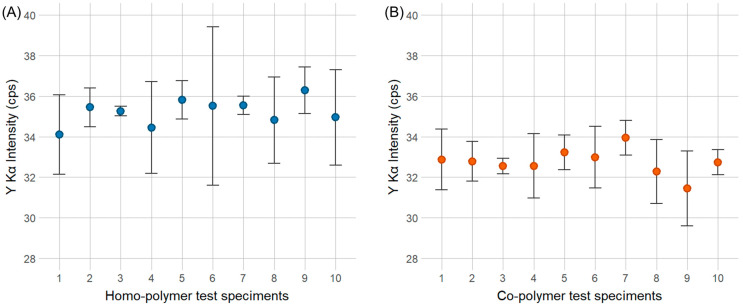
Average Kα peak height at 100 ppm Y_2_O_3_ for the homo-polymer (**A**) and co-polymer (**B**) with standard deviations of 10 test specimens. A single ANOVA test showed no significant differences in the dispersibility of marker material within the same polymer type.

The dispersibility tests for the Y_2_O_3_ marker show that the average Y Kα peak height for 10 co-polymer test specimens was 32.74 ± 1.17 (94 ± 5 ppm) and 35.22 ± 1.64 cps (102 ± 6 ppm) for 10 homo-polymer test specimens. The single ANOVA test calculated F-values of 0.779 for the co-polymer and 0.339 for the homo-polymer. The *p*-value was above the α-significance level for both polymer types, indicating no significant difference in the Y Kα peaks between samples. Therefore, it can be concluded that Y_2_O_3_ marker material was homogeneously dispersed both in homo- and co-polymer test specimens. A graphical overview of three measurement positions per sample, i.e., on both ends and in the middle of the test specimens, for the Y_2_O_3_ marker in the homo- and co-polymer is shown in Figure 7, and the data are displayed in Table S9 in the Supplementary Materials.

**Figure 8:**
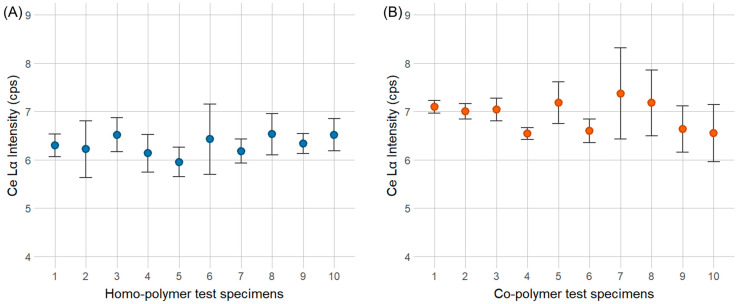
Average Lα peak height at 1000 ppm CeO_2_ for the homo-polymer (**A**) and co-polymer (**B**) with standard deviations of 10 test specimens. A single ANOVA test showed no significant differences in the dispersibility of marker material within the same polymer type.

Analog to the yttrium markers, the dispersibility tests for the CeO_2_ marker show that the average Lα peak height for 10 test specimens of the co-polymer is 69.92 ± 0.40 cps (1001 ± 130 ppm), while the average Ce Lα peak height for homo-polymer is 6.31 ± 0.38 cps (936 ± 141 ppm). A graphical overview is displayed in Figure 8 and the data can be found in Table S10 in the Supplementary Materials. The single ANOVA test calculated F-values of 1.246 for the co-polymer and 0.651 for the homo-polymer. The *p*-value was above the α-significance level for both polymer types, indicating no significant difference in the Ce Lα peaks between samples. Therefore, it can be concluded that CeO_2_ marker material was homogeneously dispersed both in homo- and co-polymer test specimens.

The results of the ANOVA test indicate that the null hypothesis (cf. Tables S11 and S12 in the Supplementary Materials) cannot be rejected. This suggests that the observed differences in marker concentrations between the samples are not statistically significant. Consequently, it can be concluded that the marker material was homogeneously distributed for both polymer types (co- and homo-polymer) and for both marker concentrations (yttrium at 100 ppm and cerium at 1000 ppm).

### 3.5. Closed-Loop Recycling Scenario

#### 3.5.1. Impacts on Material Properties

Material tests were performed to test the mechanical properties of virgin POM plastics with and without the Y_2_O_3_ marker, as well as POM plastics after the simulated recycling process with and without the Y_2_O_3_ marker. The test results (Table 3) show that the MFR and MVR of the POM plastic samples after the simulated recycling process increased compared to the virgin POM plastics independent of the Y_2_O_3_ marker or POM polymer type. Furthermore, a slight decrease in the tensile elongation factor εY (%) was observed for POM plastics after the simulated recycling process in both polymer types. The highest tensile elongation factor εY (%) was found in virgin POM co-polymer plastics with the Y_2_O_3_ marker.

#### 3.5.2. Impacts on Marker Substance Concentration

To compare and assess the impact of the simulated recycling process on the Y_2_O_3_ marker content, 30 s pXRF measurements were conducted on virgin POM homo- and co-polymer samples and samples after the simulated recycling process (see Table 4).

**Figure 9:**
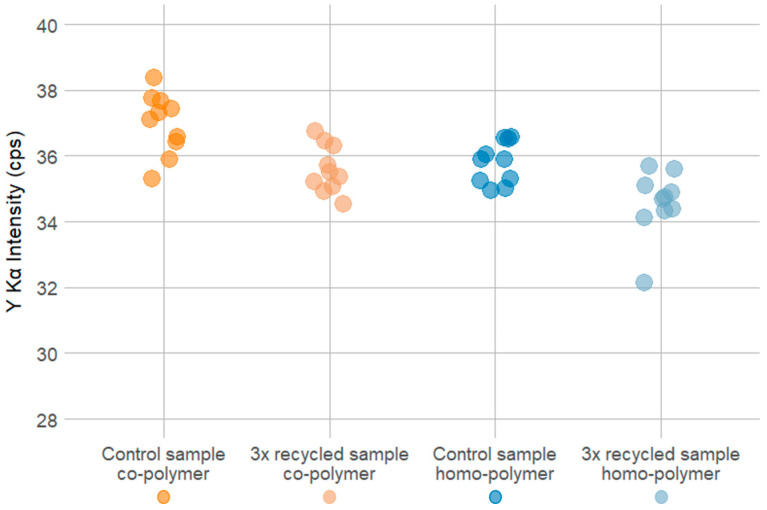
Comparison of Y_2_O_3_ content in the homo- and co-polymer samples after production of the first batch of POM plastics with marker substance (control sample) and after simulated recycling process consisting of re-melting and re-extruding three times (3× recycled samples).

For the virgin POM co-polymer control samples (before the simulated recycling process), the mean Y Kα peak height was 36.98 ± 0.93 cps (108 ± 3 ppm), while for the co-polymer, the Y Kα peak height was 35.80 ± 0.63 cps (104 ± 2 ppm). After the simulated threefold recycling process, the mean Y Kα peak height for the co-polymer decreased to 35.59 ± 0.72 cps (104 ± 2 ppm) and 34.57 ± 1.00 cps (99 ± 3 ppm) for the homo-polymer. In order to verify the statistical significance of the observed differences at α ≤ 0.05, two-sample *t*-tests for each polymer type were conducted. The two-sample *t*-test for the co-polymer resulted in a t-value of 3.75 and a *p*-value of 0.001, while the two-sample *t*-test for the homo-polymer resulted in a t-value of 3.30 and a *p*-value of 0.001. Consequently, for both the homo- and co-polymer, the decrease in Y Kα peak height is statistically significant. A graphical summary of the pXRF analyses of the POM samples before and after the simulated recycling process is shown in Figure 9. The results of the two-sample *t*-test show that after the simulated threefold recycling process, the Y Kα peak height decreases by 3.75 cps in the co-polymer and 3.30 in the homo-polymer over the three recycling cycles.

### 3.6. Thermal Treatment Scenario

#### 3.6.1. Thermogravimetric Analysis

The TGA resulted in the complete combustion of the polymer compound. The polymer marker compounds using the POM co-polymer exhibited a higher rate of disintegration, resulting in a much smaller amount of ash residue (Table S13 in the Supplementary Materials). The mass of the sample post-combustion was calculated by subtracting the weight of the empty crucible from the weight of the crucible containing the sample post-combustion. The negative value for the POM co-polymer sample post-combustion is most likely a result of the measurement inaccuracy of the balance used for weighing. The TGA curves are available in Figure S3 in the Supplementary Materials.

#### 3.6.2. Composition Analysis of Ashes

**Figure 10:**
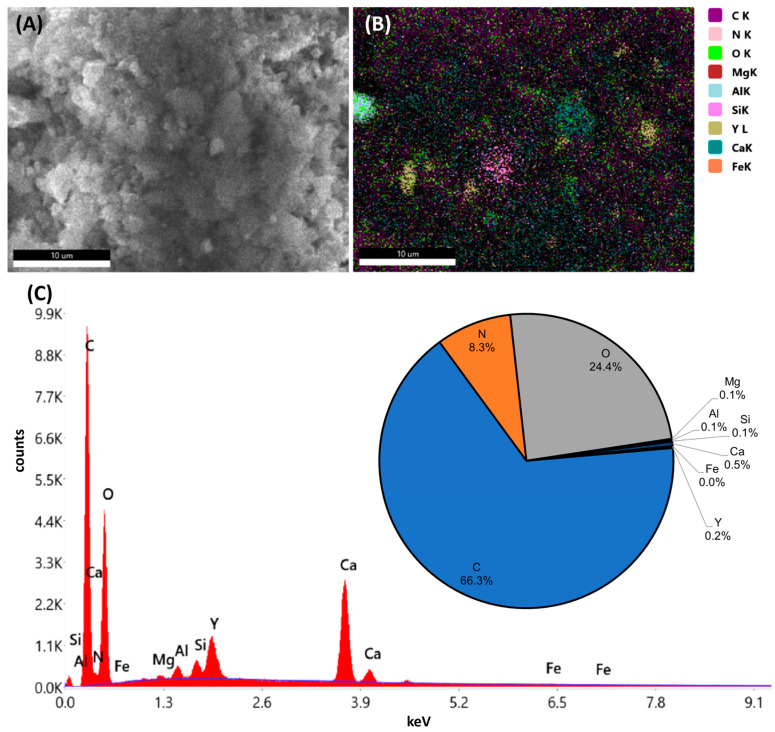
SEM/EDX measurements of the Y_2_O_3_-marked POM co-polymer ash sample at 10,000×magnification, 20 keV, and a 22 min measurement time. (**A**) SEM image, (**B**) elemental mapping, (**C**) corresponding EDX spectrum, including a pie chart depicting the atom percentages.

Morphological analysis of the incineration residues using an SEM shows that the ash consists of fine particles and bigger, brittle structures, which are an agglomeration of smaller particles as visible in Figure 10. An EDX analysis of the POM homo-polymer with and without Y_2_O_3_, as well as the POM co-polymer with and without Y_2_O_3_ oxide, were performed (Figure 10 and Figures S4–S6 in the Supplementary Materials). The analysis shows a high carbon peak in each sample, that originates from the carbon adhesive strip and carbides in the sample. The second-highest peak in most samples was oxygen, which indicates the presence of carbonates or oxides in the ash of the Y_2_O_3_-marked samples. In both ashes of the Y_2_O_3_-marked samples (Figure 10 and Figure S5 in the Supplementary Materials), yttrium could be detected, whereas in the unmarked samples (Figures S4 and S6 in the Supplementary Materials), no yttrium could be detected. The presence of yttrium could also be visualized using the elemental mapping feature, depicting regions containing yttrium in a swamp green color (Figure 10) Additionally, in contrast to the POM co-polymer samples, which display a high peak of calcium and only a tracer peak of silicon, the POM homo-polymer shows a higher peak of silicon and manganese.

Magnesium, silicon, calcium, aluminum, and iron found in the ashes are impurities within the marker material. The elevated levels of Mg, Si, and Ca can also be attributed to their presence as residuals from the polymer manufacturing process. Aluminum may also originate from abrasive impurities from the TGA crucible.

## 4. Discussion

Mechanical properties were tested according to the ISO 527-2 [22] and 179-1 [19] standards for POM homo- and co-polymers separately at the minimum and maximum marker substance concentrations, i.e., 0.1 and 1000 ppm concentrations of CeO_2_ and Y_2_O_3_, respectively. The tested POM samples with the markers were compared to the control group without marker substance, where no significant differences were found. An exception was found in the elongation at the break test and the stress at the yield test for the co-polymer, where the control sample showed considerably higher values. The minor deviation observed between the two marked co-polymer samples, as well as in the homo-polymer samples, indicates that the marker particles act as nucleating agents for POM during extrusion. This effect was already observed with different materials in POM by other authors [30,31] and was also observed in the Y_2_O_3_ homo-polymer samples during the SAXS measurements, even though the addition of CeO_2_ reduced the total crystallinity of the homo-polymer in comparison to the control group. Furthermore, the considerable increase in standard deviation for the elongation at the break of the co-polymer suggests the heightened presence of thermoplastic polyurethane (PU), a common additive incorporated into the POM co-polymer. The detrimental effect of thermoplastic polyurethane (PU) on the strength and rigidity of POM has been demonstrated in numerous studies [32,33,34]. Consequently, it can be postulated that the localized impact of PU on the elongation at the break and stress at yield behavior is considerably more pronounced than that of the marker materials, which tend to act as nucleating agents and thus enhance the crystallinity of the POM co-polymer blend.

The baseline detection, quantification, and assessment of POM marker substance dispersibility was conducted using a portable X-ray fluorescence spectrometer (pXRF). The experimental design included the determination of optimal and minimal pXRF measurement time for both Y_2_O_3_ and CeO_2_ marker substances in the POM homo- and co-polymers by an assessment of the resulting sample spectra. The baseline detection of a marker substance was determined by two factors, i.e., pXRF measurement duration and concentration of the marker in POM plastics. The evaluation of the detectability of the marker materials in POM started at a 30 s measurement time, which is in accordance with similar pXRF analysis of other plastic additives [25,26,35,36]. The minimum detectable concentration of Y_2_O_3_ was 10 ppm, with a minimum measurement time of 10 s. In comparison, the pXRF sensitivity for the CeO_2_ marker was substantially lower, with a minimal detectable CeO_2_ concentration of 1000 ppm and a measurement time of 1 s. The calculated LOD improved to approx. 440 ppm by increasing the pXRF measurement duration to 50 s. The lower pXRF device sensitivity to Ce compared to Y was also observed by Bezati et al. [12]. These results indicate that the LOD is highly dependent on the used marker materials, their concentration, and the measurement time of the spectroscopy-based detection method. It is crucial to investigate these parameters further to ensure successful implementation on an industrial scale.

To test the dispersibility of marker substance, separate sets of 10 plastic pellets for both POM homo- and co-polymers containing 100 ppm of Y_2_O_3_ markers and 1000 ppm of CeO_2_ markers were analyzed. The results showed no significant difference between plastic pellets for both marker substances, irrespective of the polymer resin. Therefore, it can be concluded that both marker substances were well dispersed in the POM matrix.

**Figure 11:**
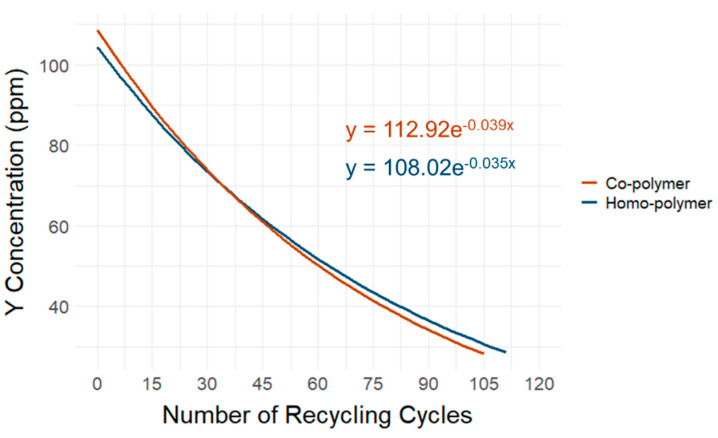
Extrapolated Y_2_O_3_ marker material loss based on the simulated recycling process in the homo- and co-polymer until the limit of detection using X-ray fluorescence was reached.

Regarding the closed-loop recycling scenario, the mechanical properties tests show that the MFR and MVR, defined by ISO 1133 [20], increased in recycled POM plastics compared to virgin POM plastics with and without the Y_2_O_3_ marker substance. A possible explanation for this deviation in the MFR and MVR is chain breaks in the semi-crystalline structure of the POM polymers caused by repeated thermal treatment [37]. Similarly, the differences in the tensile and elongation test according to ISO 527-2 [22] showed that the recycling process affected the mechanical properties of the polymer matrix. Our results indicate that the remelting processes influence the semi-crystalline structure of the polymers, regardless of whether markers or no markers were applied. We also found that re-extrusion results in a loss of marker materials. The pXRF results show statistically significant but slight losses of marker material after the recycling process. The loss of the Y_2_O_3_ marker in the POM co-polymer amounted to 3.78% after the threefold repeated extrusion process, while the loss in the POM homo-polymer reached 3.44% after the threefold repeated extrusion process. Figure 11 shows an extrapolation of Y_2_O_3_ marker substance loss based on the simulated recycling process in the homo- and co-polymer samples. The extrapolation represents a quadratic equation extension of the Y_2_O_3_ marker material loss measured as the difference between the control sample containing the marker substance and the same sample after threefold re-extrusion, simulating the recycling process. The observed reduction in the marker material was subsequently extrapolated until the LOD of the used pXRF device was reached. Based on this model, POM plastics containing 100 ppm of the Y_2_O_3_ marker substance can theoretically be recycled 109 or 105 times for the homo- and co-polymer, respectively, without adding new marker material to reach the limits of detectability of the pXRF. These results are essential for future feasibility studies, which should focus not only on economic but also on social and ecological aspects, in line with the principle of “safe and sustainable by design”.

The analysis of the simulated thermal treatment scenario showed—based on the TGA analysis—that the combustion percentage was >99%, which indicates full combustion of the polymer. The sample residues both for the homo- and co-polymers were slightly higher for samples containing the Y_2_O_3_ marker compared to the same polymer type without the marker substance. The optical analysis conducted with SEM showed high carbon content originating from the adhesive strip and carbides dispersed in fine particles and agglomerations of smaller particles. The TGA and SEM analysis results demonstrate that POM will disintegrate during conventional thermal utilization, with the markers remaining in the ash residues. This makes the material suitable for recycling, where the marker material can be recovered, for example, by leaching and precipitation methods. Further research is also required to assess the economic feasibility of this approach, as well as the social and ecological impact.

## 5. Conclusions

To summarize, both markers exhibit good dispersibility within both POM polymers without drastically changing the mechanical parameters of the polymer composite, even though some deviations within elongation at break and strain at yield to the control sample were shown. Additionally, detailed analysis using SAXS and WAXS indicates a slight difference in the degree of crystallinity within the POM homo-polymer if Y_2_O_3_ is added, which is caused by the marker acting as a nucleating agent within the polymer. Coupled with the pXRF detectability of Y_2_O_3_ in both POM polymers at 100 ppm within 1 s, using the Y_2_O_3_ as a marker substance seems feasible on an industrial scale, whereas CeO_2_ with a worse detectability of 1000 ppm at 5s does not seem suitable. Additional upscaling, including the design of a detection system for the already existing sorting systems, will need to be further elaborated.

The laboratory-scale simulation of the close-loop recycling and thermal treatment scenario revealed that the composite has a very low ratio of marker loss when kept in a recycling loop (105 to 109 recycling cycles until the LOD is reached). It also showed that multiple recycling cycles will impact the MVR/MFR of the polymer, which needs to be considered in future studies. If the marked material should be thermally utilized, the marker can be recovered from the ashes through leaching methods, allowing for the recovery of a critical raw material, like rare earth oxides. In conclusion, it can be stated that the technical feasibility of closed-loop recycling by remelting or extruding thermoplastics has been demonstrated, and this process appears to be a more effective method than thermal pre-treatment with subsequent leaching for the recovery of marker materials. Following the principle of safe and sustainable design, additional feasibility studies are required to evaluate the economic, social, and ecological impacts of tracer-based sorting concepts. This approach can potentially enhance recycling rates, particularly for engineering plastics that are predominantly incinerated.

## Figures and Tables

**Table 1:** Results of material tests following ISO 527-2 and ISO 179 for the co-polymer composites and control sample. Each test displays the arithmetic mean and standard deviation (Std).

ISO-Standard	Type of Test	Parameter	CeO_2_ 1000 ppm	CeO_2_ 0.1 ppm	Y_2_O_3_ 1000 ppm	Y_2_O_3_ 0.1 ppm	Control Sample
ISO 527-2	Tensile modulus	ET (MPa)	2660.00	2660.00	2640.00	2620.00	2590.00
		Std (MPa)	52.00	40.00	43.00	33.00	51.00
	Tensile test—stress at yield	σY (MPa)	64.70	64.50	63.10	62.80	63.80
		Std (MPa)	0.73	1.25	1.06	1.40	0.19
	Strain at yield	εY (%)	8.20	7.60	6.90	6.70	12.00
		Std (%)	1.40	2.10	0.91	1.20	0.19
	Breaking strength	σB (MPa)	64.70	63.50	63.10	62.80	58.60
		Std (MPa)	0.73	1.25	1.06	1.40	3.91
	Elongation at break	εtB (%)	8.60	8.13	7.70	7.60	24.00
		Std (%)	0.99	1.60	0.74	0.93	11.00
ISO 179-1	Notch impact strength	acN (kJ/m^2^)	16.53	16.23	15.72	15.84	16.49
		Std (kJ/m^2^)	0.72	0.83	0.31	0.56	0.79

**Table 2:** Results of material tests following ISO 527-2 and ISO 179 for the homo-polymer composites and control sample. Each test displays the arithmetic mean and standard deviation (Std).

ISO-Standard	Type of Test	Parameter	CeO_2_ 1000 ppm	CeO_2_ 0.1 ppm	Y_2_O_3_ 1000 ppm	Y_2_O_3_ 0.1 ppm	Control Sample
ISO 527-2	Tensile modulus	ET (MPa)	3190.00	3160.00	3170.00	3200.00	3120.00
		Std (MPa)	81.00	88.00	79.00	75.00	72.00
	Tensile test—stress at yield	σY (MPa)	71.20	72.40	69.80	68.70	69.90
		Std (MPa)	0.71	0.46	0.82	0.51	0.46
	Strain at yield	εY (%)	7.90	7.60	7.50	7.90	8.50
		Std (%)	1.70	1.90	1.50	1.70	1.20
	Breaking strength	σB (MPa)	71.20	72.40	69.80	68.60	69.90
		Std (MPa)	0.71	0.46	0.82	0.51	0.46
	Elongation at break	εtB (%)	7.90	8.10	7.60	7.80	8.80
		Std (%)	0.77	0.86	0.79	0.76	0.83
ISO 179-1	Notch impact strength	acN (kJ/m^2^)	12.10	12.80	11.90	13.70	13.61
		Std (kJ/m^2^)	1.30	1.62	1.92	1.63	1.74

**Table 3:** Results of material tests of the polymer samples after simulated recycling. A_R_ stands for samples that underwent the simulated recycling process. A_V_ stands for “virgin” material, and 100 ppm indicates the presence of 100 ppm of Y_2_O_3_ within the sample.

			Co-Polymer				Homo-Polymer			
			A_R_-100 ppm	A_V_-100 ppm	A_R_	A_V_	A_R_-100 ppm	A_V_-100 ppm	A_R_	A_V_
ISO 527-2	Tensile test—stress at yield	σY (MPa)	60.29	64.65	65.82	67.88	67.25	71.50	66.79	73.09
	Elongation test—yield stress	εY (%)	11.08	21.66	14.13	18.62	13.96	16.47	11.64	17.39
ISO 180	Notched impact strength	acN (kJ/m^2^)	3.61	4.56	/	/	4.11	4.30	/	/
ISO 1133	Melt mass flow rate (MFR)	(g/10 min)	24.00	16.30	21.60	14.60	22.00	16.60	21.20	14.80
	Melt volume flow rate (MVR)	(cm^3^/ 10 min)	17.02	11.56	15.32	10.35	15.38	11.61	15.04	10.35

**Table 4:** A comparison of mean Y_2_O_3_ marker values in cps and ppm in 10 sample plates of the homo-polymer, 10 sample plates of the co-polymer, and 10 sample plates of each polymer type after the simulated recycling process.

	N	Mean in cps	St Dev in cps	Mean in ppm	St Dev in ppm
Control sample co-polymer	10	36.98	0.93	108	3
Threefold recycled sample co-polymer	10	35.59	0.72	104	2
Control sample homo-polymer	10	35.80	0.63	104	2
Threefold recycled sample homo-polymer	10	34.57	1.00	99	3

## Data Availability

The dataset is available upon request from the authors.

## Supplementary Materials

The following supporting information can be downloaded at: https://www.mdpi.com/article/10.3390/polym16182591/s1. Figure S1: Five pXRF repetitions of the POM plastics containing 100 ppm of Y_2_O_3_ and 1000 ppm of CeO_2_ measurement in duration intervals of 1 s, 5 s, 10 s, 20 s, 30 s, 40 s, and 50 s in order to determine the optimal measurement time; Table S1: Mean values of five repetitions of pXRF measurements of each Y_2_O_3_ and CeO_2_ concentration in the homo- and co-polymer at a 30 s measurement time; Table S2: Thermogravimetric analysis protocol—1; Table S3: Thermogravimetric analysis protocol—2; Table S4: Thermogravimetric analysis protocol—3; Table S5: Melt mass flow rate (MFR) and melt volume flow rate (MVR) for compounds of all markers and both POM homo- and co-polymers according to ISO 1133-1; Figure S2: Correlation function analysis with the Strobl model from which the short- and long-phase dimensions and the degree of crystallinity are extracted for the POM homo-polymer (H500) with different concentrations of the Y_2_O_3_ marker; Table S6: Lamellar phase dimensions and polymer crystallinity extracted from the analysis of the Fourier-transformed scattering curve; Table S7: Summary of Dunnett’s post hoc tests and the limit of detection (LOD) to test the relationship between measuring time and concentration of Y_2_O_3_ markers; Table S8: Summary of Dunnett’s post hoc tests and the limit of detection (LOD) to test the relationship between measuring time and concentration of CeO_2_ markers; Table S9: Mean values of triplicate pXRF measurements of the Y_2_O_3_ marker within 10 test specimen of the homo-polymer and 10 test specimen of the co-polymer; Table S10: Mean values of triplicate pXRF measurements of the CeO_2_ marker within 10 test specimen of the homo-polymer and 10 test specimen of the co-polymer; Table S11: Testing the Y_2_O_3_ dispersion variance within the same polymer type and between the homo- and co-polymer types; Table S12: Testing the CeO_2_ dispersion variance between sample plates of the same polymer type; Table S13: Weights of the TGA samples before and after combustion; Figure S3: TGA curves of the sample materials used during the thermal treatment scenario; Figure S4: FEI Quanta 200 SEM/EDX Spektrum, including a pie chart with atom percentages of the POM co-polymer, at 20 kEV and 10,000× magnification; Figure S5: FEI Quanta 200 SEM/EDX Spektrum, including a pie chart with atom percentages of the POM homo-polymer with 100 ppm of Y_2_O_3_, at 20 kEV and 10,000× magnification; Figure S6: FEI Quanta 200 SEM/EDX Spektrum, including a pie chart with atom percentages of the POM homo-polymer, at 20 kEV and 10,000× magnification; Table S14: List of abbreviations.
