# Supplementary material for: Adding Rare Earth Oxide Markers to Polyoxymethylene to Improve Plastic Recycling through Tracer-Based Sorting
Source: Polymers (Basel). 2024 Sep 13;16(18):2591. doi: 10.3390/polym16182591 (PMC11435554; doi:10.3390/polym16182591)
Supplement: Supplementary file 1 [file polymers-16-02591-s001.zip › Delrin500P_MSDS.pdf]

CAMPUS® Datasheet

Delrin® 500P NC010 - POM
DuPont Engineering Polymers

Product Texts

Common features of Delrin® acetal resins include mechanical and physical properties such as high mechanical strength and rigidity, excellent fatigue and impact resistance, as well as resistance to moisture, gasoline, lubricants, solvents, and many other neutral chemicals. Delrin® acetal resins also have excellent dimensional stability and good electrical insulating characteristics. They are naturally resilient, self-lubricating, and available in a variety of colors and speciality grades.

Delrin® acetal resin typically is used in demanding applications in the automotive, domestic appliances, sports, industrial engineering, electronics, and consumer goods industries.

Delrin® 500P is a general purpose medium viscosity acetal homopolymer for injection molding. It has improved processing thermal stability, a good combination of mechanical properties, and low VOC emissions.

Rheological properties	Value	Unit	Test Standard
Melt volume-flow rate, MVR	**13**	cm³/10min	ISO 1133
Temperature	**190**	°C	ISO 1133
Load	**2.16**	kg	ISO 1133
Molding shrinkage, parallel	**2.0**	%	ISO 294-4, 2577
Molding shrinkage, normal	**1.9**	%	ISO 294-4, 2577

Mechanical properties	Value	Unit	Test Standard
Tensile modulus	**3100**	MPa	ISO 527-1/-2
Yield stress	**71**	MPa	ISO 527-1/-2
Yield strain	**17**	%	ISO 527-1/-2
Nominal strain at break	**30**	%	ISO 527-1/-2
Tensile creep modulus, 1h	**2800**	MPa	ISO 899-1
Tensile creep modulus, 1000h	**1600**	MPa	ISO 899-1
Charpy impact strength, +23°C	**300**	kJ/m²	ISO 179/1eU
Charpy impact strength, -30°C	**280**	kJ/m²	ISO 179/1eU
Charpy notched impact strength, +23°C	**9**	kJ/m²	ISO 179/1eA
Charpy notched impact strength, -30°C	**8**	kJ/m²	ISO 179/1eA
Puncture - maximum force, +23°C	**2000**	N	ISO 6603-2
Puncture energy, +23°C	**3**	J	ISO 6603-2

Thermal properties	Value	Unit	Test Standard
Melting temperature, 10°C/min	**178**	°C	ISO 11357-1/-3
Temp. of deflection under load, 1.80 MPa	**93**	°C	ISO 75-1/-2
Temp. of deflection under load, 0.45 MPa	**160**	°C	ISO 75-1/-2
Vicat softening temperature, 50°C/h 50N	**155**	°C	ISO 306
Coeff. of linear therm. expansion, parallel	**100**	E-6/K	ISO 11359-1/-2
Coeff. of linear therm. expansion, normal	**100**	E-6/K	ISO 11359-1/-2
Burning behavior at 1.5 mm nominal thickness	**HB**	class	IEC 60695-11-10
Thickness tested (1.5)	**1.5**	mm	IEC 60695-11-10
Yellow Card available	**Yes**	-	-
Burning behavior at thickness h	**HB**	class	IEC 60695-11-10
Thickness tested (h)	**0.8**	mm	IEC 60695-11-10
Yellow Card available	**Yes**	-	-
Oxygen index	**22**	%	ISO 4589-1/-2

Electrical properties	Value	Unit	Test Standard
Relative permittivity, 100Hz	**3.8**	-	IEC 62631-2-1
Relative permittivity, 1MHz	**3.8**	-	IEC 62631-2-1
Dissipation factor, 100Hz	**90**	E-4	IEC 62631-2-1

Dissipation factor, 1MHz	**90**	E-4	IEC 62631-2-1
Volume resistivity	**2E12**	Ohm*m	IEC 62631-3-1
Surface resistivity	**4E14**	Ohm	IEC 62631-3-2
Electric strength	**44**	kV/mm	IEC 60243-1
Comparative tracking index	**600**	-	IEC 60112

Other properties	Value	Unit	Test Standard
Water absorption	**1.3**	%	Sim. to ISO 62
Humidity absorption	**0.2**	%	Sim. to ISO 62
Density	**1420**	kg/m³	ISO 1183

Diagrams

Viscosity-shear rate

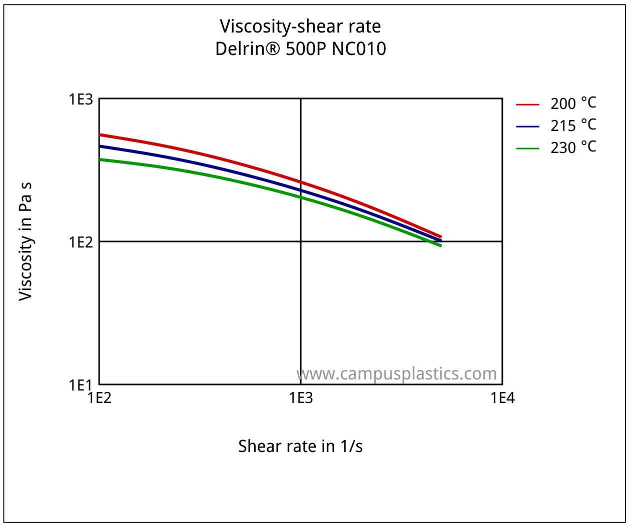

Shearstress-shear rate

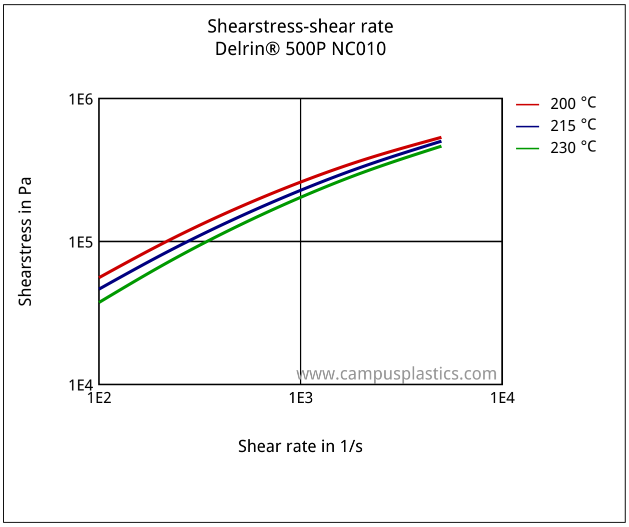

Dynamic shear modulus-temperature

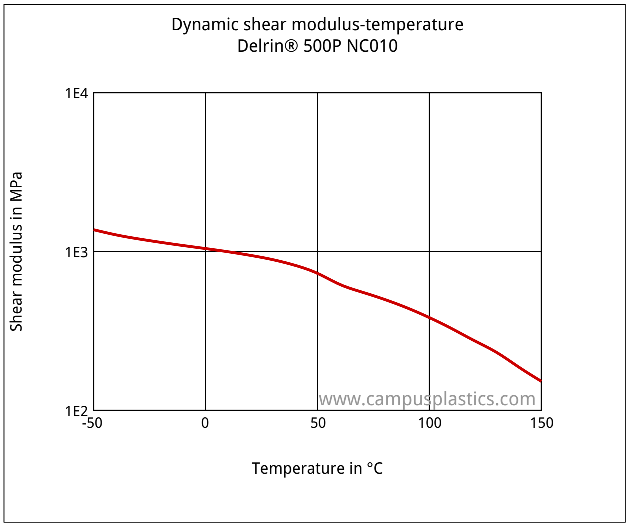

Stress-strain

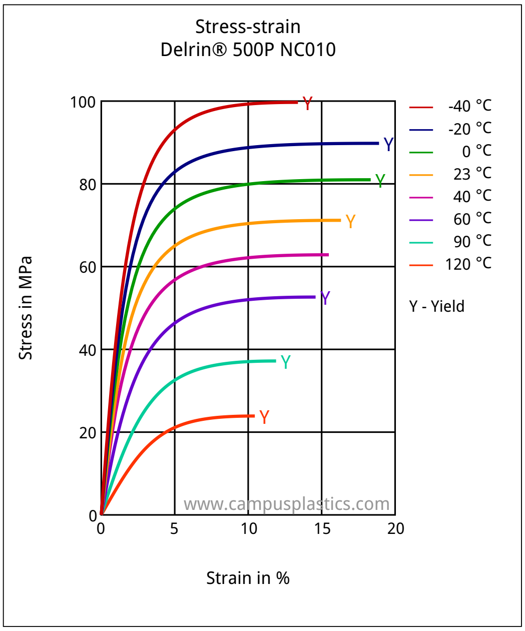

Delrin® 500P NC010 - POM
DuPont Engineering Polymers

Secant modulus-strain

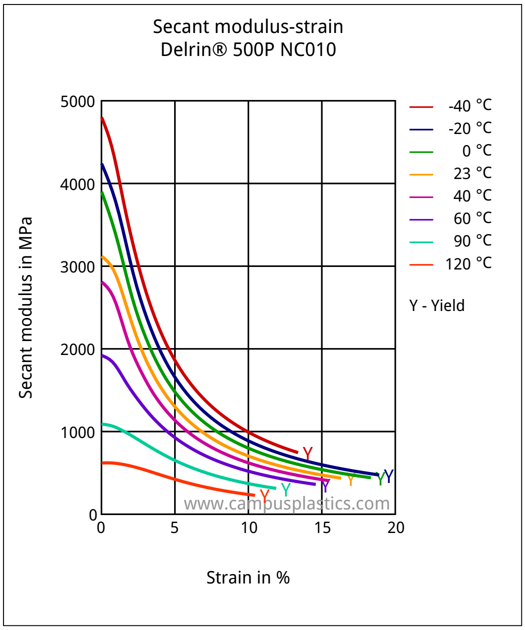

Stress-strain (isochronous) 23°C

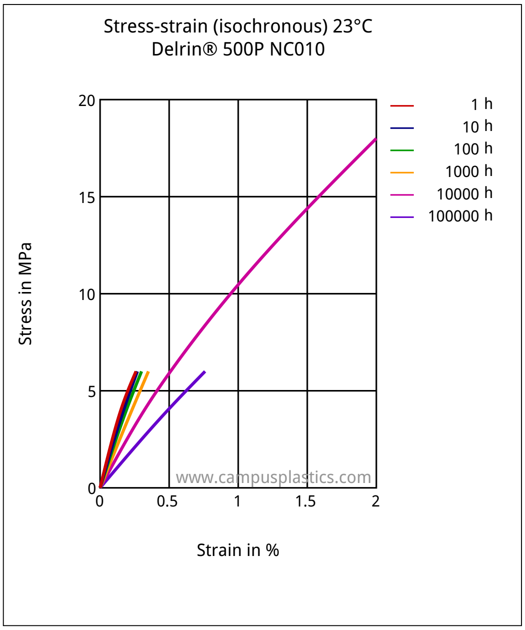

Creep modulus-time 23°C

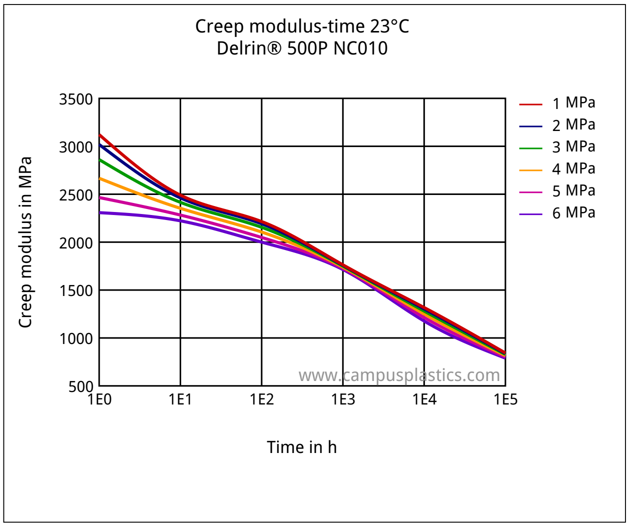

Specific volume-temperature (pvT)

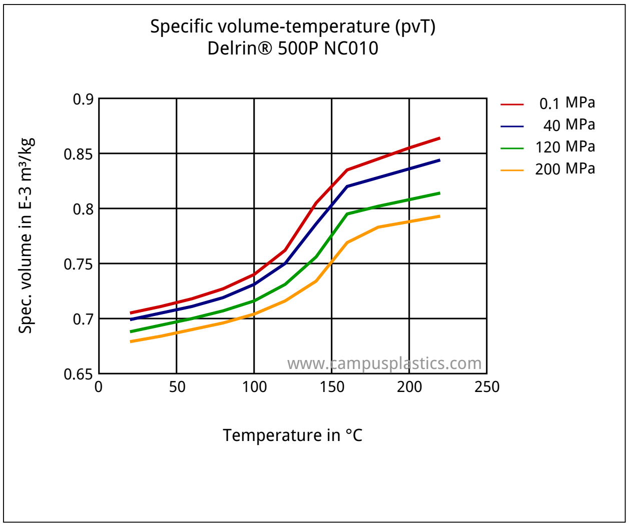

Tensile modulus-temperature

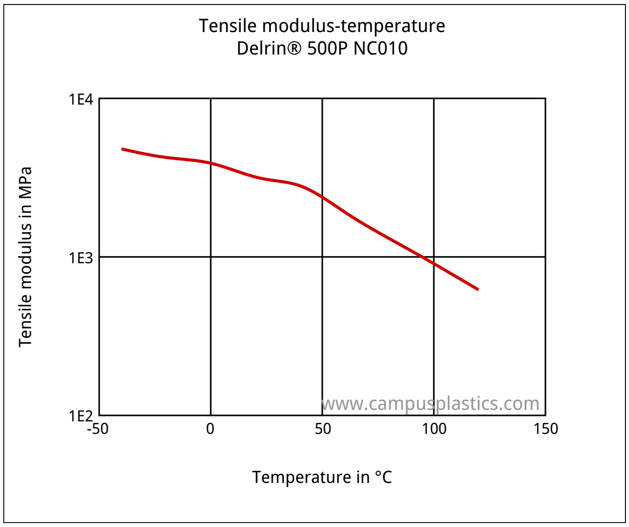

Characteristics

Processing
Injection Molding

Additives
Lubricants, Release agent

Delivery form
Pellets

Regional Availability
North America, Europe, Asia Pacific, South and Central America

Other text information

Injection molding
Drying is recommended, but not necessary for newly opened packaging stored in a dry location.

Follow the drying guidelines above in the following cases:
· If moisture is above the Processing Moisture Content recommendation,
· When a resin container is damaged,
· When the material is not properly stored in a dry place at room temperature, or
· When packaging stays open for a significant time.

Chemical Media Resistance

Acids
- Acetic Acid (5% by mass) (23°C)
- Citric Acid solution (10% by mass) (23°C)
- Lactic Acid (10% by mass) (23°C)
- Hydrochloric Acid (36% by mass) (23°C)
- Nitric Acid (40% by mass) (23°C)
- Sulfuric Acid (38% by mass) (23°C)
- Sulfuric Acid (5% by mass) (23°C)
- Chromic Acid solution (40% by mass) (23°C)

Bases
- Sodium Hydroxide solution (35% by mass) (23°C)
- Sodium Hydroxide solution (1% by mass) (23°C)

- ⛔ Ammonium Hydroxide solution (10% by mass) (23°C)

Alcohols

- 🙂 Isopropyl alcohol (23°C)
- 🙂 Methanol (23°C)
- 🙂 Ethanol (23°C)

Hydrocarbons

- 🙂 n-Hexane (23°C)
- 🙂 Toluene (23°C)
- 🙂 iso-Octane (23°C)

Ketones

- 🙂 Acetone (23°C)

Ethers

- 🙂 Diethyl ether (23°C)

Mineral oils

- 🙂 SAE 10W40 multigrade motor oil (23°C)
- ⛔ SAE 10W40 multigrade motor oil (130°C)
- ⛔ SAE 80/90 hypoid-gear oil (130°C)
- 🙂 Insulating Oil (23°C)

Standard Fuels

- 🙂 ISO 1817 Liquid 1 (60°C)
- 🙂 ISO 1817 Liquid 2 (60°C)
- 🙂 ISO 1817 Liquid 3 (60°C)
- 🙂 ISO 1817 Liquid 4 (60°C)
- 🙂 Standard fuel without alcohol (pref. ISO 1817 Liquid C) (23°C)
- 🙂 Standard fuel with alcohol (pref. ISO 1817 Liquid 4) (23°C)
- 🙂 Diesel fuel (pref. ISO 1817 Liquid F) (23°C)
- ⛔ Diesel fuel (pref. ISO 1817 Liquid F) (90°C)
- ⛔ Diesel fuel (pref. ISO 1817 Liquid F) (>90°C)
- ⛔ Diesel EN 590 (100°C)

Salt solutions

- 🙂 Sodium Chloride solution (10% by mass) (23°C)
- ⛔ Sodium Hypochlorite solution (10% by mass) (23°C)
- ⛔ Sodium Carbonate solution (20% by mass) (23°C)
- ⛔ Sodium Carbonate solution (2% by mass) (23°C)
- ⛔ Zinc Chloride solution (50% by mass) (23°C)

Other

- 🙂 Ethyl Acetate (23°C)
- ⛔ Hydrogen peroxide (23°C)
- ⛔ DOT No. 4 Brake fluid (130°C)
- ⛔ Ethylene Glycol (50% by mass) in water (108°C)
- 🙂 1% nonylphenoxy-polyethyleneoxy ethanol in water (23°C)
- 🙂 50% Oleic acid + 50% Olive Oil (23°C)
- 🙂 Water (23°C)

- Deionized water (90°C)
- Phenol solution (5% by mass) (23°C)

All data provided according to ISO 10350 for single points and ISO 11403 for multipoints.

Contact DuPont for Material Safety Data Sheet, general guides and/or additional information about ventilation, handling, purging, drying, etc.
Test temperatures are 23°C unless otherwise stated.

DuPont™, the DuPont Oval Logo, and all products, unless otherwise noted, denoted with ™, or ® are trademarks, service marks or registered trademarks of affiliates of DuPont de Nemours, Inc. © 2021 DuPont de Nemours, Inc. All rights reserved.

The information provided in this data sheet corresponds to our knowledge on the subject at the date of its publication.
This information may be subject to revision as new knowledge and experience becomes available.
The information set forth herein is furnished free of charge and is based on technical data that DuPont believes to be reliable and falls within the normal range of properties. It is intended for use by persons having technical skill, at their own discretion and risk. This data should not be used to establish specification limits nor used alone as the basis of design. Handling precaution information is given with the understanding that those using it will satisfy themselves that their particular conditions of use present no health or safety hazards. Since conditions of product use and disposal are outside our control, we make no warranties, express or implied, and assume no liability in connection with any use of this information. As with any product, evaluation under end-use conditions prior to specification is essential. Nothing herein is to be taken as a license to operate or a recommendation to infringe on patents.
DuPont advises you to seek independent counsel for a freedom to practice opinion on the intended application or end-use of our products.

CAUTION: DO NOT USE DUPONT MATERIALS IN MEDICAL APPLICATIONS INVOLVING IMPLANTATION IN THE HUMAN BODY OR CONTACT WITH INTERNAL BODY FLUIDS OR TISSUES UNLESS THE MATERIAL HAS BEEN PROVIDED FROM DUPONT UNDER A WRITTEN CONTRACT THAT IS CONSISTENT WITH DUPONT POLICY REGARDING MEDICAL APPLICATIONS AND EXPRESSLY ACKNOWLEDGES THE CONTEMPLATED USE.

For further information, please contact your DuPont representative. You may also request a copy of DuPont POLICY Regarding Medical Applications... H-50103-5 and DuPont CAUTION Regarding Medical Applications... H-50102-5.
