# Supplementary material for: Adding Rare Earth Oxide Markers to Polyoxymethylene to Improve Plastic Recycling through Tracer-Based Sorting
Source: Polymers (Basel). 2024 Sep 13;16(18):2591. doi: 10.3390/polym16182591 (PMC11435554; doi:10.3390/polym16182591)
Supplement: Supplementary file 1 [file polymers-16-02591-s001.zip › HOSTAFORMC13021_MSDS.pdf]

CAMPUS® Datasheet

HOSTAFORM® C 13021 - POM
Celanese

Celanese
The chemistry inside innovation™

Product Texts

Chemical abbreviation according to ISO 1043-1: POM Molding compound ISO 29988- POM-K, M-GNR, 04-002 POM copolymer Easy flowing Injection molding type for precision molded parts and thin-walled molded parts with high rigidity, hardness and toughness; good chemical resistance to solvents, fuel and strong alkalis as well as good hydrolysis resistance; high resistance to thermal and oxidative degradation. Monomers and additives are listed in EU-Regulation (EU) 10/2011 FDA compliant according to 21 CFR 177.2470 UL-registration for all colours and a thickness more than 1.5 mm as UL 94 HB, temperature index UL 746 B electrical 110 °C, mechanical 90 °C. Burning rate ISO 3795 and FMVSS 302 < 75 mm/min for a thickness more than 1 mm. Ranges of applications: automotive engineering, precision engineering, electric and electronical industry, domestic appliances. FDA = Food and Drug Administration (USA) UL = Underwriters Laboratories (USA) FMVSS = Federal Motor Vehicle Safety Standard (USA)

Rheological properties	Value	Unit	Test Standard
Melt volume-flow rate, MVR	**12**	$cm^3/10min$	ISO 1133
Temperature	**190**	°C	ISO 1133
Load	**2.16**	kg	ISO 1133
Molding shrinkage, parallel	**2.0**	%	ISO 294-4, 2577
Molding shrinkage, normal	**1.8**	%	ISO 294-4, 2577

Mechanical properties	Value	Unit	Test Standard
Tensile modulus	**2900**	MPa	ISO 527-1/-2
Yield stress	**65**	MPa	ISO 527-1/-2
Yield strain	**9**	%	ISO 527-1/-2
Nominal strain at break	**28**	%	ISO 527-1/-2
Tensile creep modulus, 1h	**2500**	MPa	ISO 899-1
Tensile creep modulus, 1000h	**1300**	MPa	ISO 899-1
Charpy impact strength, +23°C	**200**	kJ/m^2	ISO 179/1eU
Charpy impact strength, -30°C	**200**	kJ/m^2	ISO 179/1eU
Charpy notched impact strength, +23°C	**6.5**	kJ/m^2	ISO 179/1eA
Charpy notched impact strength, -30°C	**6**	kJ/m^2	ISO 179/1eA

Thermal properties	Value	Unit	Test Standard
Melting temperature, 10°C/min	**166**	°C	ISO 11357-1/-3
Temp. of deflection under load, 1.80 MPa	**106**	°C	ISO 75-1/-2
Coeff. of linear therm. expansion, parallel	**110**	E-6/K	ISO 11359-1/-2
Burning behavior at 1.5 mm nominal thickness	**HB**	class	IEC 60695-11-10
Thickness tested (1.5)	**1.5**	mm	IEC 60695-11-10
Burning behavior at thickness h	**HB**	class	IEC 60695-11-10
Thickness tested (h)	**3.0**	mm	IEC 60695-11-10
Yellow Card available	**Yes**	-	-

Electrical properties	Value	Unit	Test Standard
Relative permittivity, 100Hz	**4**	-	IEC 62631-2-1
Relative permittivity, 1MHz	**4**	-	IEC 62631-2-1
Dissipation factor, 100Hz	**20**	E-4	IEC 62631-2-1
Dissipation factor, 1MHz	**50**	E-4	IEC 62631-2-1
Volume resistivity	**1E12**	Ohm*m	IEC 62631-3-1
Surface resistivity	**1E14**	Ohm	IEC 62631-3-2
Electric strength	**35**	kV/mm	IEC 60243-1
Comparative tracking index	**600**	-	IEC 60112

HOSTAFORM® C 13021 - POM
Celanese

Other properties	Value	Unit	Test Standard
Water absorption	**0.65**	%	Sim. to ISO 62
Humidity absorption	**0.2**	%	Sim. to ISO 62
Density	**1410**	kg/m³	ISO 1183

Diagrams

Viscosity-shear rate

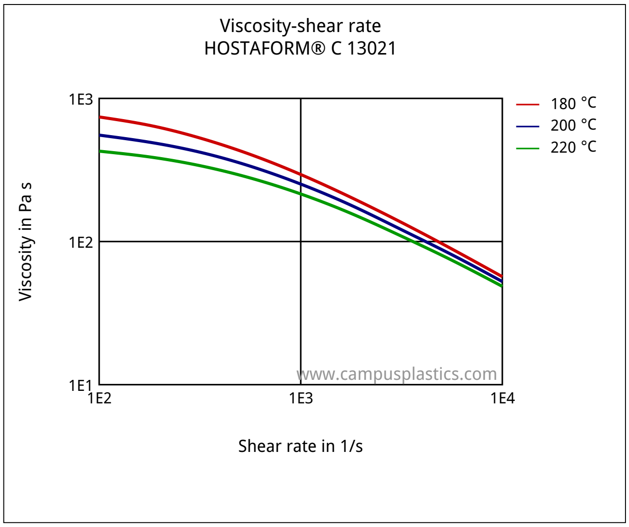

Shearstress-shear rate

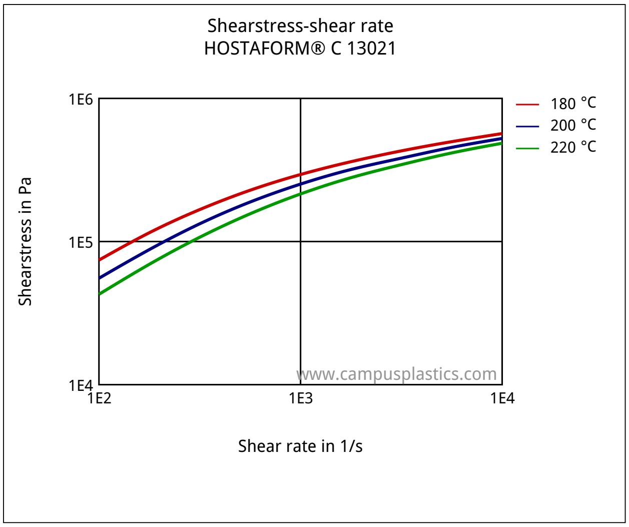

Stress-strain

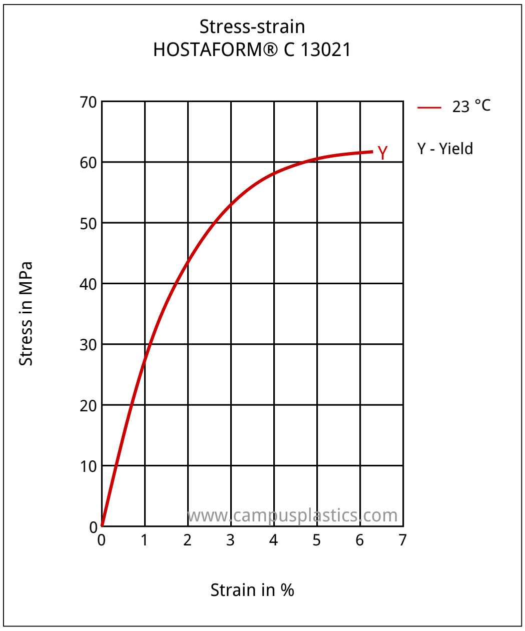

Secant modulus-strain

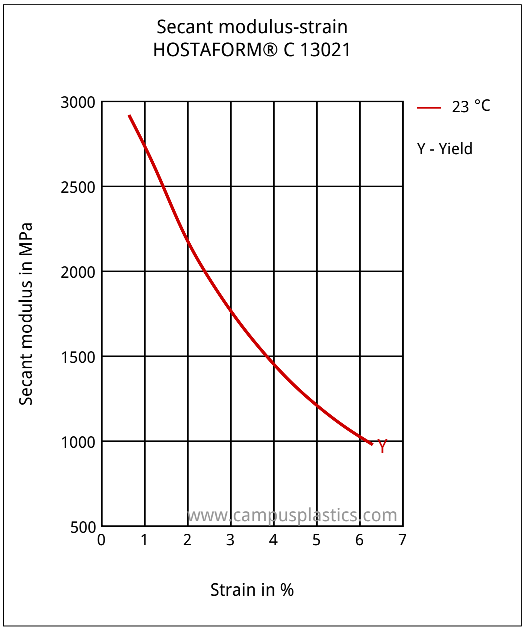

HOSTAFORM® C 13021 - POM
Celanese

Specific volume-temperature (pvT)

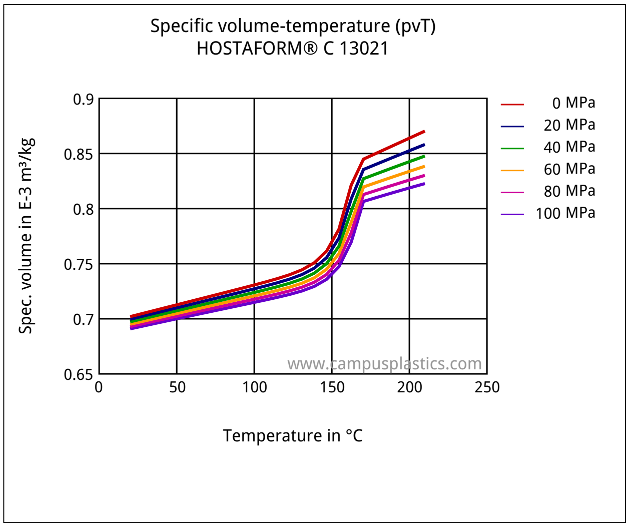

Characteristics

Processing
Injection Molding

Additives
Release agent

Delivery form
Pellets

Regional Availability
North America, Europe, Asia Pacific, South and Central America

Other text information

Injection molding

Preprocessing

General drying is not necessary due to low moisture absorption of the resin.

In case of bad storage conditions (water contact or condensed water) the use of a recirculating air dryer (100 to 120 °C / max. 40 mm layer / 3 to 6 hours) is recommended.

Max. Water content 0,2 %

Processing

Standard injection moulding machines with three phase (15 to 25 D) plasticating screws will fit.

Postprocessing

Conditioning e.g. moisturizing is not necessary.

NOTICE TO USERS: Values shown are based on testing of laboratory test specimens and represent data that fall within the standard range of properties for natural material. These values alone do not represent a sufficient basis for any part design and are not intended for use in establishing maximum, minimum, or ranges of values for specification purposes. Colorants or other additives may cause significant variations in data values. Properties of molded parts can be influenced by a wide variety of factors including, but not limited to, material selection, additives, part design, processing conditions and environmental exposure. Other than those products expressly identified as medical grade (including by MT® product designation or otherwise), Celanese's products are not intended for use in medical or dental implants. Regardless of any such product designation, any determination of the suitability of a particular material and part design for any use contemplated by the users and the manner of such use is the sole responsibility of the users, who must assure themselves that the

material as subsequently processed meets the needs of their particular product or use. To the best of our knowledge, the information contained in this publication is accurate; however, we do not assume any liability whatsoever for the accuracy and completeness of such information. The information contained in this publication should not be construed as a promise or guarantee of specific properties of our products. It is the sole responsibility of the users to investigate whether any existing patents are infringed by the use of the materials mentioned in this publication. Moreover, there is a need to reduce human exposure to many materials to the lowest practical limits in view of possible adverse effects. To the extent that any hazards may have been mentioned in this publication, we neither suggest nor guarantee that such hazards are the only ones that exist. We recommend that persons intending to rely on any recommendation or to use any equipment, processing technique or material mentioned in this publication should satisfy themselves that they can meet all applicable safety and health standards. We strongly recommend that users seek and adhere to the manufacturer’s current instructions for handling each material they use, and entrust the handling of such material to adequately trained personnel only. Please call the telephone numbers listed for additional technical information. Call Customer Services for the appropriate Materials Safety Data Sheets (MSDS) before attempting to process our products.
© 2024 Celanese or its affiliates. All rights reserved. Celanese®, registered C-ball design and all other trademarks identified herein with ®, TM, SM, unless otherwise noted, are trademarks of Celanese or its affiliates. Fortron is a registered trademark of Fortron Industries LLC. KEPITAL is a registered trademark of Korea Engineering Plastics Company, Ltd.
