# Supplementary material for: Adding Rare Earth Oxide Markers to Polyoxymethylene to Improve Plastic Recycling through Tracer-Based Sorting
Source: Polymers (Basel). 2024 Sep 13;16(18):2591. doi: 10.3390/polym16182591 (PMC11435554; doi:10.3390/polym16182591)
Supplement: Supplementary file 1 [file polymers-16-02591-s001.zip › Markers-in-POM-for-TBS_Supplementary informations_proofread.pdf]

Appendix A. Supplementary Information

Manuscript Title: Adding rare earth oxide markers to polyoxymethylene to improve plastic recycling through tracer-based sorting

Authors: Aleksander Jandric, Christoph Olscher*, Christian Zafiu, Robert Lielacher, Christoph Lechner, Andrea Lassenberger and Florian Part

Number of Pages (not including cover page): 11

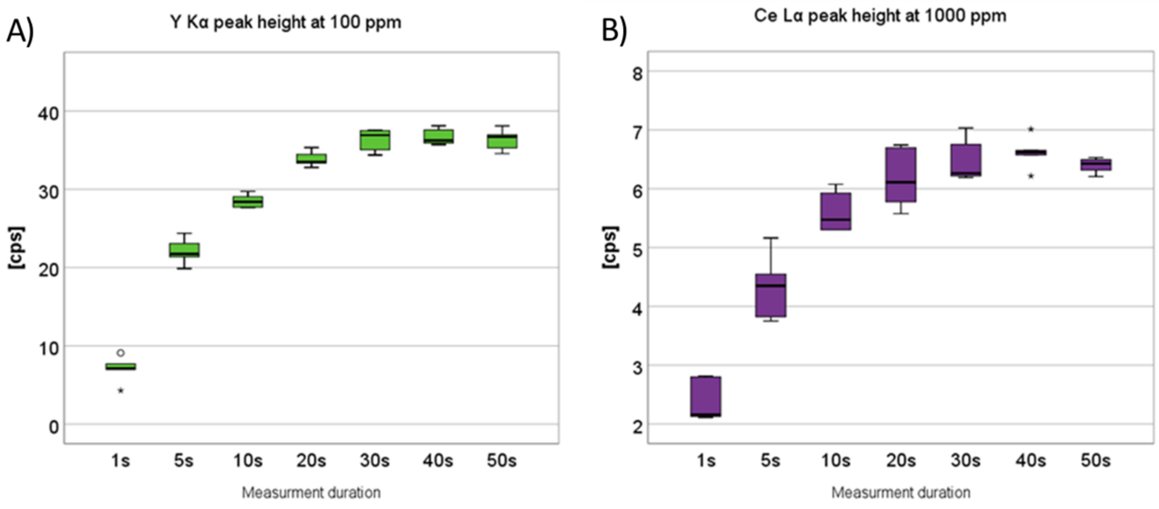

Figure S1: Five pXRF repetitions of the POM plastics containing 100 ppm of Y_2O_3 and 1000 ppm of CeO_2 measurement in duration intervals of 1 s, 5 s, 10 s, 20 s, 30 s, 40 s, and 50 s in order to determine the optimal measurement time.

Table S1: Mean values of five repetitions of pXRF measurements of each Y_2O_3 and CeO_2 concentration in the homo- and copolymer at a 30 s measurement time.

Marker concentration	Y Kα peak in homo-polymer	Y Kα peak in co-polymer	Ce Lα peak in homo-polymer	Ce Lα peak in co-polymer
[ppm]	[cps]			
0	9.63	7.68	3.86	3.88
0.1	9.64	7.72	3.76	4.01
1	9.78	8.15	3.91	3.69
10	10.37	9.83	3.77	3.92
100	34.04	36.28	4.13	3.70
1000	258.93	275.81	6.92	6.49

Table S2: Thermogravimetric analysis protocol - 1

Instrument:	NETZSCH STA 409 C/CD	TG TG RANGE	50000	mg
Project		GAS 1 MFC Range	313	ml/min
Filename:	POM-H500+Y2O3-100_mod_gross1_20	GAS 2 MFC Range	248	ml/min
Date/time:	17.11.2022 11:38:27 (UTC+1)	GAS 3 MFC Range	313	ml/min
End date/time:	17.11.2022 15:31:31 (UTC+1)	Sample identiy	POM-H500+Y2O3-100_mo	
Laboratory:	IVET	Sample name:	POM-H500+Y2O3-100_mo	
Operator:	RO	Sample mass:	793.49	mg
Mode:	TG	Crucible:	DSC/TG pan Al2O3	
Measurement Type:	Probe	Crucible mass:	0	mg
Temp. calib.:	TCALZERO.TCX	Material	leer	
crucible:	DSC/TG pan Al2O3	Sample determination mode	Manual	

Table S3: Thermogravimetric analysis protocol - 2

Furnace:		SKIMMER 4			Furnace TC:	S
Sample carrier:		TG HIGH RG 2			Sample TC:	S
Measurement end:		Normal End			M. Range:	50000 mg
Gas 1:	ARGON	Flow:			predefined	
Gas 2:	AIR(20/20)	Flow:			predefined	
Gas 3:	ARGON	Flow:			predefined	
Gas 1 MFC:	ARGON	Flow range:	312.50	ml/min	predefined	
Gas 2 MFC:	OXYGEN	Flow range:	248.00	ml/min	predefined	
Gas 3 MFC:	ARGON	Flow range:	312.50	ml/min	predefined	

Table S4: Thermogravimetric analysis protocol - 3

Temperature levels												
NUM	Mode	Temp °C	HR (K/min)	Acq. Rate (pts/min)	Duration (hh:mm)	STC	G1	G2	G3	G1:Ar	G2:O2	G3:Ar
--	Stand-by: heating	34.00	1.00			0	0	0	0	60.0	31.0	60.0
--	Stand-by: isothermal	34.00			00:10	0	0	0	0	60.0	31.0	60.0
1	Dynamic	500.00	2.00	40.00	03:53	0	0	0	0	60.0	31.0	60.0
--	Emergency	550.00					0	0	0			10.0

Table S5: Melt mass flow rate (MFR) and melt volume flow rate (MVR) for compounds of all markers and both POM homo- and copolymers according to ISO 1133-1. 0* stands for “blank” POM without markers.

	POM homo-polymer		POM co-polymer	
	Melt mass flow rate (MFR)			
ppm	CeO_2	Y_2O_3	CeO_2	Y_2O_3
1000	15.7	16.6	15.3	16.6
100	16.1	16.3	14.6	16.3
10	15.2	15.9	15.9	15.9
1	17.1	15.8	14.7	15.8
0.1	15.8	16.5	14.6	16.5
0*	14.8		14.6	
	Melt volume flow rate (MVR)			
1000	10.98	11.26	10.85	11.77
100	11.26	11.61	10.35	11.56
10	10.63	11.75	11.28	11.28
1	11.96	11.47	10.43	11.21
0.1	11.05	10.98	10.33	11.21
0*	10.35		10.35	

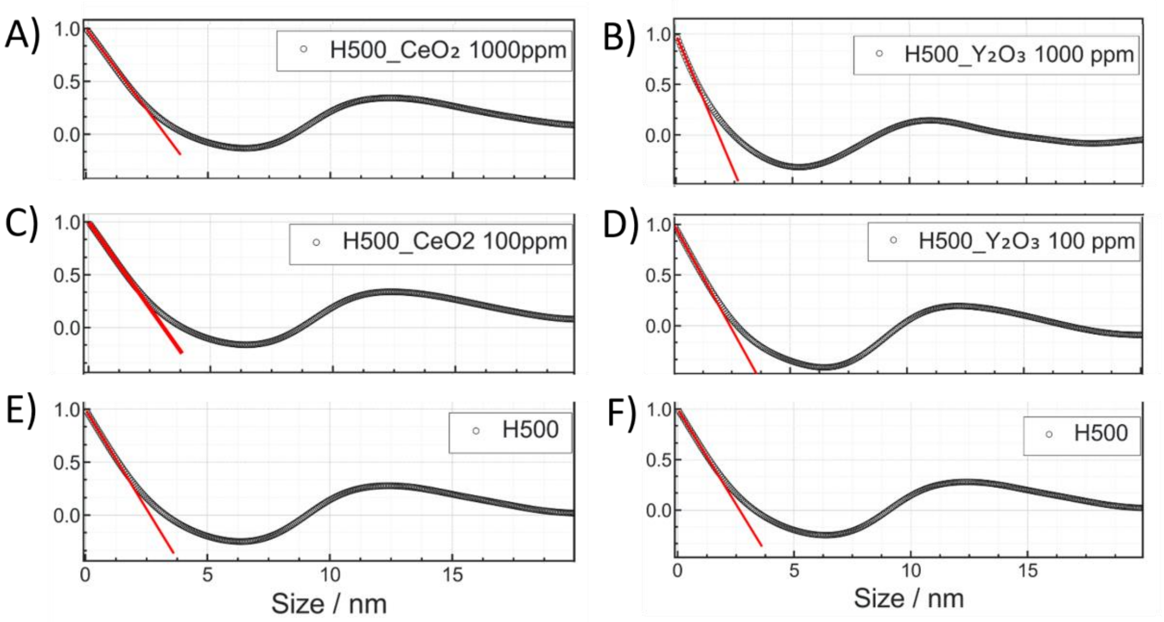

Figure S2: Correlation function analysis with the Strobl model from which the short- and long-phase dimensions and the degree of crystallinity are extracted for the POM homo-polymer (H500) with different concentrations of the Y_2O_3 marker.

Table S6: Lamellar phase dimensions and polymer crystallinity extracted from the analysis of the Fourier-transformed scattering curve

	Short period / nm	Long period / nm	Degree of crystallinity
Homopolymer	12.4	3.1	0.20
Homopolymer + CeO_2 100 ppm	12.4	3.4	0.14
Homopolymer + CeO_2 1000 ppm	12.4	3.7	0.11
Homopolymer + Y_2O_3 100 ppm	12.2	3.4	0.29
Homopolymer + Y_2O_3 1000 ppm	10.9	2.4	0.24

Table S7: Summary of Dunnett's post hoc tests and the limit of detection (LOD) to test the relationship between measuring time and concentration of Y_2O_3 marker.

Y_2O_3	Summary of Dunnett's post hoc tests						
Marker concentration	1 s	5 s	10 s	20 s	30 s	40 s	50 s
0.1 ppm	0.47	0.82	0.86	0.80	0.77	0.95	0. 86
1 ppm	0.69	0.81	0.79	0.68	0.40	0.97	0.91
10 ppm	0.35	0.08	0.04	0.00	0.00	0.00	0.00
100 ppm	0.00	0.00	0.00	0.00	0.00	0.00	0.00
1,000 ppm	0.00	0.00	0.00	0.00	0.00	0.00	0.00
LOD (cps)	3.36	7.01	7.35	8.23	8.37	9.17	8.33
LOD (ppm)	16	10	5	3	4	4	3

Table S8: Summary of Dunnett's post hoc tests and the limit of detection (LOD) to test the relationship between measuring time and concentration of CeO_2 markers

CeO_2	Summary of Dunnett's post hoc tests						
Marker concentration	1 s	5 s	10 s	20 s	30 s	40 s	50 s
0.1 ppm	0.46	0.85	0.80	0.89	0.51	0.95	0.95
1 ppm	0.24	0.77	0.98	0.99	0.99	0.95	0.96
10 ppm	0.25	0.91	0.52	0.96	0.75	0.74	0.99
100 ppm	0.45	0.79	0.63	0.39	0.99	0.84	1.00
1,000 ppm	0.04	0.00	0.00	0.00	0.00	0.00	0.00
LOD (cps)	2.89	5.74	4.42	4.74	4.55	4.92	3.89
LOD (ppm)	2,318	2,197	439	389	282	366	442

Table S9: Mean values of triplicate pXRF measurements of the Y_2O_3 marker within 10 test specimen of the homo-polymer and 10 test specimen of the co-polymer.

Y_2O_3	Homo-polymer				Co-polymer			
Sample pellet	Mean in cps	St dev in cps	Mean in ppm	St dev in ppm	Mean in cps	St dev in cps	Mean in ppm	St dev in ppm
1	32.87	1.50	95	6	34.11	1.96	98	7
2	32.78	0.97	95	4	35.45	0.96	103	4
3	32.54	0.39	94	2	35.26	0.24	102	1
4	32.55	1.59	94	6	34.45	2.27	99	8
5	33.22	0.85	96	3	35.82	0.95	104	4
6	32.98	1.53	95	6	35.51	3.90	103	15
7	33.95	0.86	99	3	35.55	0.45	103	2
8	32.28	1.58	93	6	34.82	2.12	100	8
9	31.45	1.84	89	7	36.29	1.15	106	4
10	32.74	0.62	94	2	34.95	2.36	101	9
**Mean**	**32.74**	**1.17**	**94**	**5**	**35.22**	**1.64**	**102**	**6**

Table S10: Mean values of triplicate pXRF measurements of CeO_2 marker within 10 test specimen of the homo-polymer and 10 test specimen of the co-polymer.

CeO_2	Homo-poylmer				Co-polymer			
Sample	Mean in cps	St dev in cps	Mean in ppm	St dev in ppm	Mean in cps	St dev in cps	Mean in ppm	St dev in ppm
1	7.10	0.13	1,057	43	6.30	0.23	931	86
2	7.00	0.16	1,028	52	6.22	0.59	902	217
3	7.04	0.23	1,041	75	6.52	0.35	1,012	131
4	6.54	0.12	878	40	6.14	0.39	870	145
5	7.18	0.43	1,086	139	5.95	0.31	803	113
6	6.60	0.24	897	78	6.43	0.72	978	268
7	7.37	0.94	1,147	304	6.18	0.25	887	93
8	7.18	0.68	1,085	220	6.53	0.43	1,017	158
9	6.64	0.48	910	156	6.33	0.21	944	78
10	6.55	0.59	883	191	6.52	0.33	1,012	123
**Mean**	**6.92**	**0.40**	**1,001**	**130**	**6.31**	**0.38**	**936**	**141**

Table S11: Testing the Y_2O_3 dispersion variance within the same polymer type and between the homo- and co-polymer types.

Statistical test	Sample description	p-value	Hypothesis
Single ANOVA	10 co-polymer plates with 100 ppm Y_2O_3	0.64	H_0 cannot be rejected → no significant difference of marker substance between sample plates
Single ANOVA	10 homo-polymer plates with 100 ppm Y_2O_3	0.95	H_0 cannot be rejected → no significant difference of marker substance between sample plates

Table S12: Testing the CeO_2 dispersion variance between sample plates of the same polymer type

Statistical test	Sample description	p-value	Hypothesis
Single ANOVA	10 co-polymer plates with 1,000 ppm CeO_2	0.32	H_0 cannot be rejected → no significant difference of marker substance between sample plates
Single ANOVA	10 homo-polymer plates with 1,000 ppm CeO_2	0.74	H_0 cannot be rejected → no significant difference of marker substance between sample plates

Table S13: Weights of the TGA samples before and after combustion

Sample	Crucible empty (mg)	Sample pre comb. (mg)	Crucible + Sample pre comb. (mg)	Crucible + Sample post comb. (mg)	Sample post comb. (mg)
POM homo-polymer 100 ppm Y_2O_3	2,836.86	793.49	3,630.35	2,839.25	2.39
POM homo-polymer	2,840.74	855.76	3,696.53	2,843.00	2.26
POM co-polymer 100 ppm Y_2O_3	2,865.19	706.07	3,571.25	2,865.97	0.78
POM co-polymer	2,866.66	863.38	3,730.05	2,865.97	-0.69

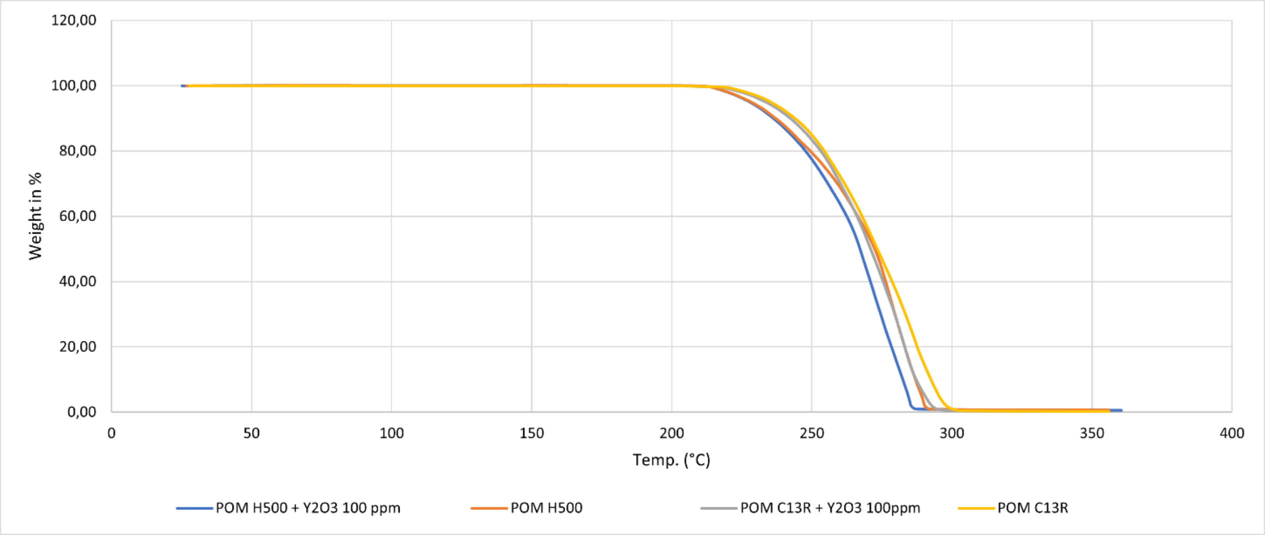

Figure S3: TGA curves of the sample materials used during the thermal treatment scenario

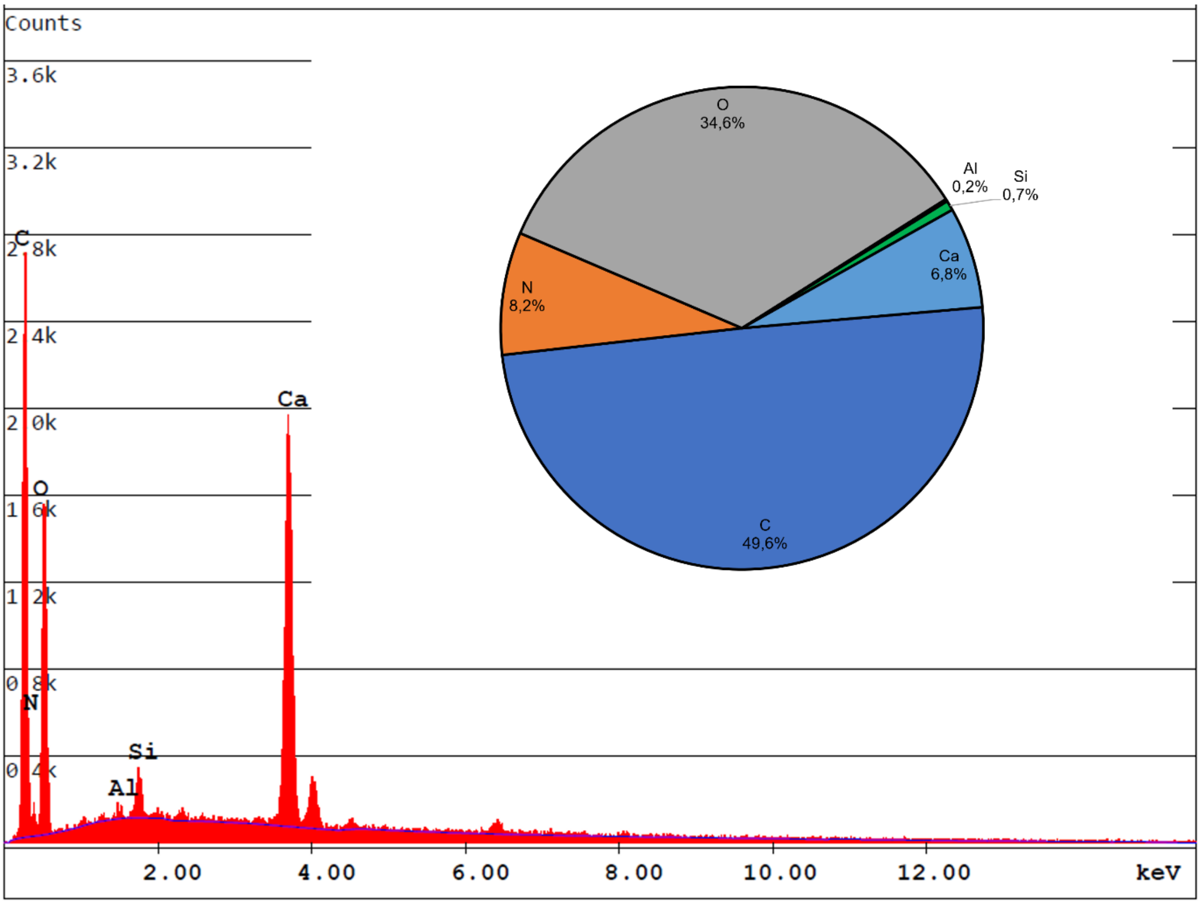

Figure S4: FEI Quanta 200 SEM/EDX Spektrum including a pie chart with atom percentages of the POM co-polymer, at 20 kEV and 10,000× magnification

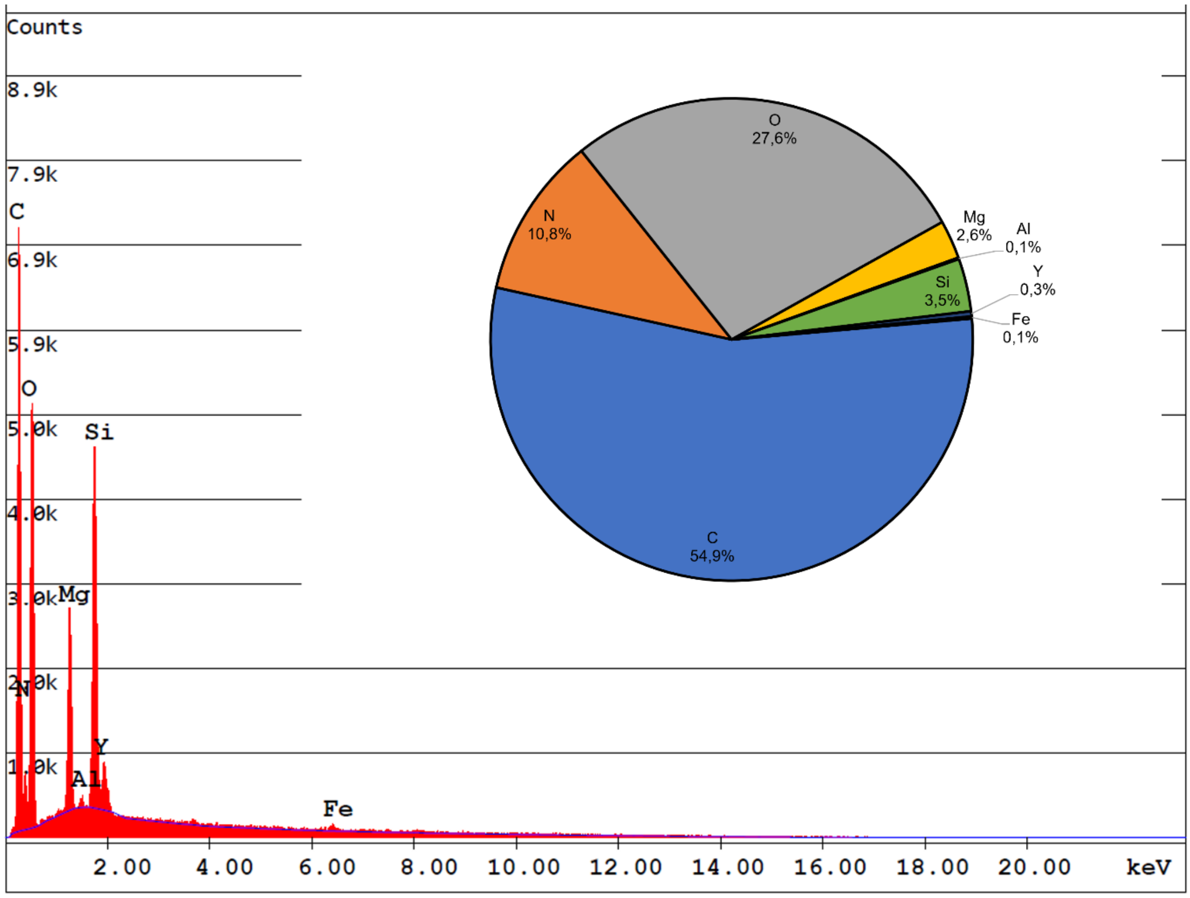

Figure S5: FEI Quanta 200 SEM/EDX Spektrum including a pie chart with atom percentages of the POM homo-polymer with 100 ppm of Y_2O_3, at 20 kEV and 10,000× magnification

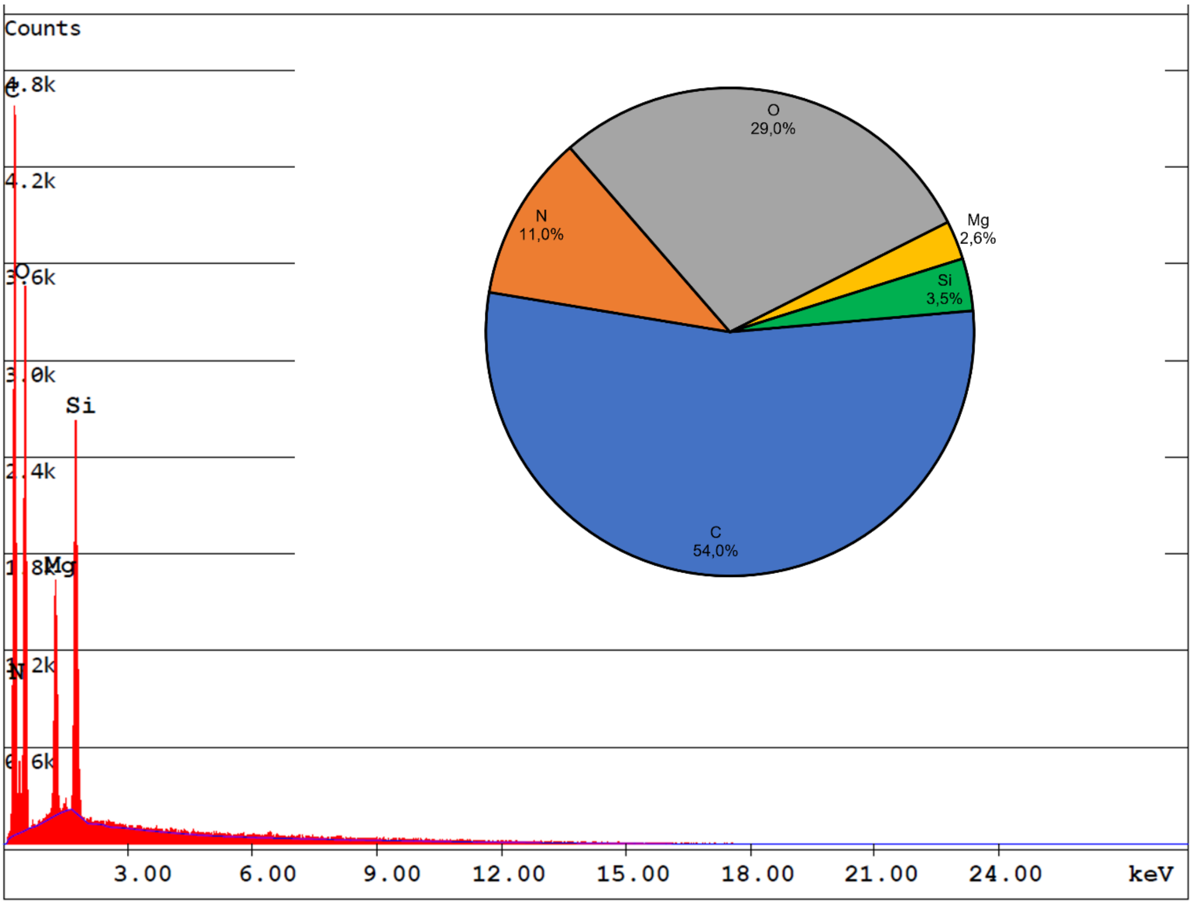

Figure S6: FEI Quanta 200 SEM/EDX Spektrum including a pie chart with atom percentages of the POM homo-polymer, at 20 kEV and 10,000× magnification.

Table S14: List of abbreviations

ANOVA	Analysis of variance
cps	Counts per second
DF	Degrees of freedom
EDX	Electron dispersive x-ray (analysis)
H_0	Null hypothesis
HDPE	High-density polyethylene
IR	Infra-red
LDPE	Low-density polyethylene
LOD	Limit of Detection
MFR	Melt mass-flow rate
MVR	Melt volume-flow rate
NIR	Near infra-red
PET	Polyethylene terephthalate
POM	Polyoxymethylene
PP	polypropylene
PS	polystyrene
PVC	Polyvinyl chloride
pXRF	Portable x-ray fluorescence
ROI	Region of interest
RT	Room temperature
SAXS	Small-angle x-ray scattering
SEM	Scanning electron microscope
Std	Standard deviation
TGA	Thermogravimetric analysis
TPU	Thermoplastic urethane
TRL	Technology readiness level
WAXS	Wide-angle x-ray scattering
